# Cancer proteome and metabolite changes linked to SHMT2

**DOI:** 10.1371/journal.pone.0237981

**Published:** 2020-09-09

**Authors:** Jiefei Tong, Jonathan R. Krieger, Paul Taylor, Rick Bagshaw, Jae Kang, Swathi Jeedigunta, Leanne E. Wybenga-Groot, Wen Zhang, Heba Badr, Shideh Mirhadi, Nhu-An Pham, Étienne Coyaud, Man Yu, Ming Li, Michael Cabanero, Brian Raught, Jason T. Maynes, Cynthia Hawkins, Ming Sound Tsao, Michael F. Moran

**Affiliations:** 1 Program in Cell Biology, The Hospital for Sick Children, Toronto, Canada; 2 SPARC BioCentre, The Hospital for Sick Children, Toronto, Canada; 3 Program in Molecular Medicine, The Hospital for Sick Children, Toronto, Canada; 4 Department of Molecular Genetics, University of Toronto, Toronto, Canada; 5 Princess Margaret Cancer Centre, University Health Network, Toronto, Canada; 6 Department of Laboratory Medicine and Pathobiology, University of Toronto, Toronto, Canada; 7 Department of Medical Biophysics, University of Toronto, Toronto, Canada; Columbia University, UNITED STATES

## Abstract

Serine hydroxymethyltransferase 2 (SHMT2) converts serine plus tetrahydrofolate (THF) into glycine plus methylene-THF and is upregulated at the protein level in lung and other cancers. In order to better understand the role of SHMT2 in cancer a model system of HeLa cells engineered for inducible over-expression or knock-down of SHMT2 was characterized for cell proliferation and changes in metabolites and proteome as a function of SHMT2. Ectopic over-expression of SHMT2 increased cell proliferation *in vitro* and tumor growth *in vivo*. Knockdown of SHMT2 expression *in vitro* caused a state of glycine auxotrophy and accumulation of phosphoribosylaminoimidazolecarboxamide (AICAR), an intermediate of folate/1-carbon-pathway-dependent *de novo* purine nucleotide synthesis. Decreased glycine in the HeLa cell-based xenograft tumors with knocked down SHMT2 was potentiated by administration of the anti-hyperglycinemia agent benzoate. However, tumor growth was not affected by SHMT2 knockdown with or without benzoate treatment. Benzoate inhibited cell proliferation *in vitro*, but this was independent of SHMT2 modulation. The abundance of proteins of mitochondrial respiration complexes 1 and 3 was inversely correlated with SHMT2 levels. Proximity biotinylation *in vivo* (BioID) identified 48 mostly mitochondrial proteins associated with SHMT2 including the mitochondrial enzymes Acyl-CoA thioesterase (ACOT2) and glutamate dehydrogenase (GLUD1) along with more than 20 proteins from mitochondrial respiration complexes 1 and 3. These data provide insights into possible mechanisms through which elevated SHMT2 in cancers may be linked to changes in metabolism and mitochondrial function.

## Introduction

The conversion of serine into glycine catalyzed by serine hydroxymethyltransferases (SHMT1 and SHMT2) releases one carbon units and is a nexus of cancer metabolism and cell regulation. SHMT1 and SHMT2 proteins are largely localized to the cytosol and mitochondria, respectively, but isoforms are also found in the nucleus during S-phase and function in *de novo* thymidylate biosynthesis [1]. A cytosolic isoform of SHMT2 also functions as a scaffolding subunit of a complex that deubiquitinates IFNAR1 and regulates interferon responses [2]. SHMT2 therefore impinges several aspects of cancer cell regulation and metabolism, including catalyzing the folate-dependent production of precursors for DNA synthesis and methylation (e.g. purines, pyrimidines, S-adenosylmethionine), and the production of NADPH that further supports glutathione production, important in redox balance and growth under hypoxic conditions [3,4].

Tumors may become “addicted” to deregulated serine biosynthesis and glycine cleavage pathways [5–8]. Serine biosynthesis is fueled by the glycolysis intermediate 3-phosphoglycerate (3PG) and requires a glutamate-derived amino group, generated by the conversion of glutamate into alpha-ketoglutarate (αKG) by phosphoserine aminotransferase (PSAT1). These requirements are reflected in the high rates of consumption of glucose and glutamine (as a source of glutamate), which epitomise transformed cells. Serine is produced from 3PG by the sequential action of phosphoglycerate dehydrogenase (PHGDH), PSAT1, and phosphoserine phosphatase (PSPH). Our multi-omics analysis of aggressive, early-stage non-small cell lung cancer (NSCLC) revealed PHGDH, PSAT1 and especially SHMT2 as highly up-regulated compared to normal lung as a consequence of proteome remodelling, and showed PSPH gene amplification linked with EGFR [9]. SHMT2 expression is up-regulated downstream of oncogenic transcription factors including NRF2 in lung cancer [10] and MYC in breast cancer [8]. Our results [9] and subsequent studies confirmed that elevated SHMT2 levels are associated with tumor aggressiveness and poor prognosis [10–13].

SHMT2 is linked to several aspects of metabolism important to cancer cell survival and proliferation. SHMT2 knockdown slowed or stopped the proliferation of transformed cells when cultured in glycine free medium, and is directly linked to glycine-dependent, transformed cell proliferation rates [6]. The products of serine breakdown by SHMT2 include glycine and 5, 10-methylene tetrahydrofolate (THF), which upon conversion to 10-formyl THF generates NADPH. SHMT2-dependent NADPH production maintains redox balance to support tumor cell survival and proliferation [4]. SHMT2 regulates the activity or expression of proteins that enable tumor cell survival under hypoxia or nutrition deficiencies [7,13–15]. Increased SHMT2 decreases cytosolic levels of serine and phosphoribosylaminoimidazolesuccinocarboxamid (SAICAR), two activators for pyruvate kinase 2 (PKM2), which allows redistribution of glycolytic carbons in support of cancer cell proliferation [3]. Recently SHMT2 was shown to regulate mitochondrial respiration complex I protein expression by epigenetic [14–16] or non-epigenetic [17] mechanisms. Decreased SHMT2 expression has shown variable effects *in vivo* ranging from no effect to dramatic inhibition of tumor growth [3,4,6,18]. These inconsistencies may reflect cell type specific differences in SHMT2-dependent processes and metabolites such as the expression of glycine decarboxylase (GLDC) and glycine transporters that regulate intracellular glycine levels [19]. SHMT2 has also been shown to affect cell proliferation under certain stress scenarios such as in tumor ischemic regions [3]. Hence, SHMT2 is linked to several aspects of metabolism important to cancer cell survival and proliferation but is not established as a cancer drug target.

In this study mass spectrometry (MS) was used to measure changes in proteome and metabolite profiles as a function of SHMT2 expression in a model cell line grown in culture or when implanted in a mouse model. Changes in the level of SHMT2 protein expression *in vitro* and *in vivo* affected the expression of mitochondrial respiration complex proteins and metabolites including its substrates/products serine and glycine. The anti-hyperglycinemia drug benzoate was effective in modulating glycine levels in vitro and *in vivo*. However, SHMT2 knockdown with or without benzoate treatment did not inhibit xenograft tumor growth. Benzoate inhibited cell proliferation *in vitro*, but this effect was independent of SHMT2 expression levels. These findings extend our understanding of the diverse contributions of SHMT2 to the cancer phenotype and regulation of mitochondrial function.

## Materials and methods

### Cells, constructs, and reagents

HeLa and HEK-293 cells were purchased from American Type Culture Collection. The NSCLC cell line LPC43 was established from a patient-derived xenograft (PDX) tumor (PHLC178/ADC6) with informed patient consent and Research Ethics Board approval (REB# 09–0510, University Health Network, Toronto, ON, Canada) [20]. LPC43 and HeLa cells were maintained in RPMI 1640 supplemented with 10% fetal bovine serum. HEK293 cells were grown in DMEM with 10% bovine serum. All cells were cultured at 37°C in a 5% CO2 humidified incubator unless otherwise specified.

A plasmid containing the mouse SHMT2 open reading frame cDNA was obtained from SPARC BioCentre (Hospital for Sick Children, Toronto). To generate a C-terminal Flag-tagged construct, the SHMT2 cDNA was amplified by PCR using specific primers encoding the epitope tag, and subcloned into pcDNA3.1-Myc/His (Invitrogen) or pcDNA5 FRT/TO MCS-BirA^R118G^. The SHMT2 open reading frame was also subcloned into tetracycline (Tet) inducible pcDNA5/FRT/TO expression vector (Invitrogen) by Gateway cloning. For shRNA silencing, the pLKO-1-Puro lentiviral expression vector was obtained from Dr. Benjamin Neel (Princess Margaret Cancer Centre, Toronto, Canada). All expression vectors were verified by DNA sequencing.

Antibodies used were goat polyclonal anti-SHMT2 (sc-25064) from Santa Cruz Biotechnology Inc. (SCB), mouse monoclonal anti-FLAG M2 from Sigma-Aldrich, and rabbit antibodies to polyADP(ribose) polymerase (PARP) (Cat#5625) from Cell Signaling Technology Inc. (CST). Other antibodies were purchased from CST or SCB. The mitochondrion dye marker MitoTracker Red CMXRos was purchased from CST. All other reagents were purchased from Sigma-Aldrich unless otherwise specified.

### Generation of inducible SHMT2 over-expression and knockdown cell lines

For stable Tet-inducible over-expression of SHMT2, pcDNA5/FRT/TO- or pcDNA5 FRT/TO MCS-BirA^R118G^ –SHMT2-Flag vectors were transfected into T-Rex-HeLa cells (Invitrogen), and selected with 200 μg/ml hygromycin B. For stable SHMT2-Flag over-expression, pcDNA3-SHMT2-Flag was transfected into HEK-293 and individual colonies were selected in medium with G418 (800 μg/ml). In order to make cells with isopropyl β-D-1-thiogalactopyranoside (IPTG)-inducible shRNA to SHMT2, two DNA oligonucleotides (5’CCGGCGGAGAGTTGTGGACTTTATACTCGAGTATAAAGTCCACAA CTCTCCGTTTTTG-3’ and 5’AATTCAAAAACGGAGAGTTGTGGACTTTATACTCGAGTAT AAAGTCCACAACTCTCCG-3’) were annealed and cloned into the vector pLKO-1-Puro. The corresponding lentivirus was produced and used to infect target cells following standard protocols at SPARC BioCentre (Hospital for Sick Children). The stably transduced SHMT2 over-expression or knockdown cells were selected by cell growth in medium containing 200 μg/ml hygromycin B or 2μg/mL puromycin, respectively. The engineered HeLa cells with Tet-inducible over-expression of SHMT2 and IPTG-inducible shRNA against SHMT2, named as HeLa-SHMT2-shSHMT2 or simplified as HeLa-Ss, were maintained in RPMI medium with hygromycin and puromycin.

### Cell proliferation, soft agar colony formation assay and xenograft growth

For SHMT2 over-expression, tetracycline was added to HeLa-Ss cells on day 0, and for SHMT2 knock down, HeLa-Ss cells were treated with IPTG for 7 d and then split on day 0 with fresh addition of IPTG.

For proliferation assays, 2000 HeLa-Ss cells or 5000 LPC43 cells were seeded per well in 96-well plates on day 0. Cells were switched into RPMI medium supplemented with 10% dialyzed fetal bovine serum (FBS) containing a full set of amino acids or lacking glycine as indicated. Plates were washed with phosphate-buffered saline (PBS), fixed and stained on the indicated days with 0.2% crystal violet, 2% ethanol, and then washed with distilled water. Crystal violet was solubilized in 1% sodium dodecylsulfate (SDS), and measured at 570 nm [21].

For soft agar colony formation assay, HeLa-Ss were cultured with a base layer of 0.4% agarose and an assay layer of 0.3% agarose (Becton Dickinson, BD). HeLa-Ss cells were seeded at 1E4 cells per well in 6-well plates. Plates were scanned and analyzed after 21 d as described [21].

*In vivo* experiments were carried out at the University Health Network (UHN) animal facility (Toronto, Canada) by using animal protocols approved by the Animal Care Committee and which conform to the Canadian Council on Animal Care. Mice were kept in a pathogen-free environment on a standard 12 h day, 12 h night cycle, and were fed a standard diet of sterilized pellet and water *ad libitum*. Non-obese diabetic severely combined immune deficient (NOD-SCID) mice at 4–6 weeks of age were obtained from the UHN in-house breeding program. For xenograft studies, 1E6 HeLa-Ss cells were implanted subcutaneously in the right flank of NOD-SCID nude mice, with 5 mice per control (Cont), SHMT2-Up and -Down groups, and with 9 mice per sodium benzamide (Benz) and Benz-Down groups [22]. Animals were maintained humanely by technicians in the UHN animal facility according to mandated institute and government guidelines, with no more than 5 mice per cage. Drinking water with 1% glucose, 0.4% sucrose, and 2 mg/ml doxycycline, or 10 mM IPTG and 1% glucose was provided to control, SHMT2-Up and SHMT2-Down mice, respectively. For Benz treated mice, sodium benzoate was added to 1% in drinking water [23]. Tumor volumes were measured by using the formula for a hemi-ellipsoid: volume = 0.5236 × length × width × height. Mice were scarified immediately if tumors caused deterioration of health, or tumor diameter reached 1.5 cm. At the end of assay, mice were sacrificed using isoflurane, and tumor specimens collected and flash frozen in liquid nitrogen [22].

### Affinity purification and western blotting

In order to immune-precipitate SHMT2 protein complexes, HEK293 cells or HeLa variants were lysed in NP-40 buffer(1% NP40, 0.5% Deoxycholate, 50 mM Tris pH 7.5, 150 mM NaCl, 10% glycerol, 25 mM NaF, 1 mM EDTA, plus protease inhibitors (Thermo Pierce Protease Inhibitor Tablet, A32963) and phosphatase inhibitors (Thermo Pierce Phosphatase Inhibitor Tablet, 88662), and subject to Immunoaffinity purification by using immobilized anti-FLAG M2 affinity agarose beads as described previously in Tong *et al*. [20]. For affinity purification mass spectrometry (AP-MS), isolated immune complexes were eluted with 0.15% trifluoroacetic acid (TFA), and processed for downstream proteomic analysis or western blot analysis as described [20].

### Identification of SHMT2 protein interactions by proximity-dependent biotin identification and MS

In order to identify proteins proximal and/or physically associated with SHMT2 in living cells, the BioID method was employed [24]. Biotinylated proteins were affinity purified by using immobilized streptavidin, followed by trypsin digestion, MS, and bioinformatics analysis for peptide and protein identifications essentially as described previously [25].

For proteomic analysis of tandem mass tag (TMT)-labeled samples, 2% SDS lysis buffer (100 mM HEPES pH 7.3 and 2% SDS, 50 mM NaCl, 10 mM TCEP((Tris (2-Carboxyethyl) phosphine Hydrochloride), 40 mM CAA (Chloroacetamide), and complete protease inhibitor (same as in NP40 buffer)) was used to quickly lyse cells from cell culture or from tumor samples by heating at 95°C for 15 min [26]. Protein (50 μg) was precipitated by using methanol-chloroform and then digested with a trypsin/Lys-C mixture (1 μg, Promega Cat#V5073) [27]. Digested peptides (25 μg) were labeled with 10-plex TMT reagents according to the manufacturer’s instructions (ThermoFisher Scientific). Ten labelled samples as indicated were pooled and fractionated by using the Pierce high pH kit (Pierce Cat#84868). Eight fractions of each pooled TMT sample were then analyzed by using an Orbitrap Fusion Lumos Tribrid Mass Spectrometer (Thermo Fisher Scientific) [26,27].

### Immunostaining and confocal microscopy

Hela cells were grown on coverslips for 24 h with tetracycline induction, and then fixed with freshly prepared 3.7% paraformaldehyde, permeabilized, and then probed by targeted antibodies as indicated. Confocal microscopy was performed by using a Zeiss LSM 510 META laser-scanning microscope (Carl Zeiss Inc., Thornwood, NY). Additional details on experimental protocols were as described previously [20].

### Glycolysis and mitochondria respiration

Extracellular acidification rate (ECAR) as a measure of glycolysis and oxygen consumption rate (OCR) as a measure of mitochondrial respiration were analyzed by using an XFe96 Extracellular Flux Analyzer (Seahorse Bioscience). For SHMT2 knockdown or over-expression conditions, HeLa-Ss cells were grown in medium with IPTG addition for 6 d or Tet for 3 d before cell counting and seeding. Cells (2E4) were seeded onto a Seahorse XF96 cell culture microplate coated with poly-L-lysine the day before the experiment and analyzed according to the manufacturer’s protocol. For mitochondrion stress test, final concentrations of oligomycin, FCCP, and rotenone/antimycin A were 1 μM, 0.5 μM and 0.5 μM respectively. For glycolysis analysis, final concentrations of glucose, oligomycin and 2-deoxyglucose (DG) were 10 mM, 1 μM and 50 mM, respectively. Protein amounts were measured by BCA protein assay (Thermo Fisher Scientific) and used to normalize OCR and ECAR results. Data were viewed and analyzed in Wave Desktop version 2.2 (Seahorse Bioscience). The XF Glycolysis Stress Test Report Generator and the XF Mito Stress Test Report Generator (Seahorse Bioscience) were used to automatically calculate glycolysis and mitochondrial respiration parameters from Wave data. Three independent experiments of each test were performed with a minimum of 6 replicate wells per condition [15].

### Analysis of metabolites by MS

For cell metabolite analysis cells were plated in 6-well plates, grown under the indicated treatment conditions, and washed with cold 0.9% NaCl before metabolite extraction. Cellular metabolites were extracted with cold 400 μl extraction buffer (40% acetonitrile, 40% methanol and 20% water) per well for 6-well plates as described previously [28,29]. For tumor samples, cold 200 μl extraction buffer/10 mg wet tissue was added to samples followed by brief sonication and storage at -80°C overnight. Metabolites in extraction buffer were dried and analyzed by targeted LC-MS/MS by using selected reaction monitoring mass spectrometry (SRM). SRM transitions are listed in S1 Table in S1 Data.

MS analysis of metabolites was completed by using an Ultimate 3000 HPLC coupled to a TSQ Vantage triple quadrupole MS instrument (Thermo Fisher Scientific). Reverse-phase (RP) or hydrophilic interaction liquid chromatography (HILIC) were used to separate metabolites as described previously [30]. Metabolite levels in 4-to-9 biological replicates were normalized to protein content. Samples were analyzed using an Inertsil ODS-3 C18, 3 μm, 150 mm x 4.6 mm I.D. column (GL Sciences, Cat#5020–01771) for RP-LC/MS analysis, and ZIC-pHILIC, 5 μm, 150 mm x 4.6 mm I.D. column (Canadian LifeScience, Cat#SQ2812-155) for HILIC/MS analysis. The standard mobile phase, A = 0.1% formic acid in water and B = 0.1% formic acid in 95% acetonitrile, was used for RPLC in electrospray ionization (ESI) positive/negative mode. The mobile phase, A = 20 mM ammonium carbonate (pH9.2) and B = 100% acetonitrile, was used for HILIC in both ESI positive and negative modes. The chromatographic gradient for RPLC was as follows: 0–12 min: increase linearly from 5% B to 60% B; 12–20.5 min: increase linearly to 80% B; 12.5–18 min: hold at 80% B; 18–19 min: decrease linearly to 5%B; 19–25 min: hold at 5% B. The chromatographic gradient for HILIC was as follows: 0–4 min: decrease linearly from 80% B to 65% B; 4–13 min: decrease linearly to 40% B; 13–18 min: hold at 40% B; 18–21 min: increase linearly to 80% B. The flow rate was 300 μL/min and the sample injection volume was 10 μl.

For metabolite data analysis, Skyline software from the MacCoss laboratory was used to extract targeted metabolite measurements [31] (https://skyline.gs.washington.edu/). The alignment of targeted metabolite peaks was manually checked with standards and final data were normalized by median for each sample by using Excel software. For heat map analysis, normalized metabolites in arbitrary units extracted from Skyline were compared to maximal intensities of corresponding metabolites and expressed in a range from 0 to 1. Values are means of 4-to-9 biological replicates for each sample.

Raw LC-MS data in mzXML format of all metabolites are accessible via the MetaboLights repository (www.ebi.ac.uk/metabolights) under accession number MTBLS405.

### Mitochondrion analysis

Mitochondrion staining and quantification was performed by using MitoTracker Red CMXRos (Invitrogen, M7510) according to the manufacture’s protocol. For coupling with immunostaining, MitoTracker Red CMXRos (100 nM) was incubated with live cells for 30 min and then immunostaining was carried out as described above. For quantification, 1E4 cells/well were seeded on 96 cell plates one day before the experiment, incubated with MitoTracker Red CMXRos (1 μM) for 30 min, and then lysed by addition of 100 μl 1% SDS followed by agitation for 15 min. Fluorescent signals were measured at an excitation wavelength of 579 nm and emission wavelength of 612 nm. Cell number in a parallel plate was counted and used for normalization.

### Glutathione analysis

Glutathione levels in live cells were measured by monochlorobimane (MCB) assay [32]. Briefly, 10 μl MCB (100 μM) was added to a well of a 96 well plate with 100 μl medium, incubated for 30 min in a cell culture incubator after which fluorescence was measured by using an excitation wavelength of 380 nm and emission wavelength of 485 nm.

### MS analysis of polypeptides

MS sample preparation was performed essentially as described previously [9,25,33]. Tryptic peptides were concentrated and purified on homemade C18 columns or C18 StageTips (Thermo Fisher Scientific) before liquid chromatography-tandem MS. Peptides were separated by reverse-phase chromatography by using a nanoflow UPLC system (Thermo Fisher Scientific) with a linear gradient. Ultra performance liquid chromatography (UPLC) was coupled online to an Orbitrap Elite or Q-Exactive or Lumos mass spectrometer (Thermo Fisher Scientific). Peptide ions were fragmented by collision-induced dissociation (CID).

Analysis of TMT labeled peptide fractions was carried out on an Orbitrap Lumos MS platform (Thermo Scientific). Data acquisition was carried out using a data-dependent acquisition method with multi-notch synchronous precursor selection and MS3 scanning for TMT tags [26].

For AP-MS and BioID datasets, raw MS files acquired from the MS analyzer were processed by using MaxQuant software (version 1.3.0.5) according to the standard workflow [34]. MS/MS spectra were searched against the UniProt human proteome (release 2017) by using the Andromeda search engine [35]. For statistical evaluation of data, a false discovery rate of 0.01 was set for peptide and protein identification. Protein label free quantification (LFQ) intensity was chosen as the quantitative value representing protein abundance and used for calculations of protein differential expression. Protein LFQ intensities across different samples were first normalized by the intensities of the SHMT2 bait protein. The logarithmic ratio of protein intensities between two samples and the negative logarithmic p-values of the Welch’s t-test obtained from biological triplicates between two samples were calculated for Volcano plot analysis by using Perseus software (perseus-framework.org/) [36]. Cytoscape version 3.2.0 [37] was used for visualization of protein interaction networks.

For TMT labeled samples, raw MS files acquired from the MS analyzer were processed by Proteome Discoverer Software (ver. 2) and Scaffold software (Proteome Software, Inc., Oregon, USA). The same reference sample, a mixture of 18 samples, was used for two sets of TMT samples. The relative amount of proteins compared to the reference sample were extracted from Proteome Discoverer Software (Thermo, USA), converted to relative fold change compared to control and then transferred to log2 values. For cell culture samples, SHMT2 UP and Down values are relative to control, non-induced cells; i.e. Cont values were set to 1. For tumor samples the Up and Down values are presented relative to the average of three Cont tumor samples. Perseus software was used for statistical and cluster analyses. In the heatmap white is set as the median value. Proteins identified by TMT methods required more than 95% probability in peptide and protein identification.

MS proteomics data described in this paper have been deposited to the ProteomeXchange Consortium (http://www.proteomexchange.org) via the PRIDE partner repository with the dataset identifier PXD004629 for BioID data, and PXD004954 for TMT data.

### Statistical analysis

The significance of difference between groups was determined by the Student’s t-test in Excel (Microsoft) or by using Perseus tools available in the MaxQuant environment.

## Results

### Elevated serine/glycine/1-carbon metabolism proteins in lung tumors and models, and establishment of SHMT2 dependent HeLa cell lines

**Fig 1:**
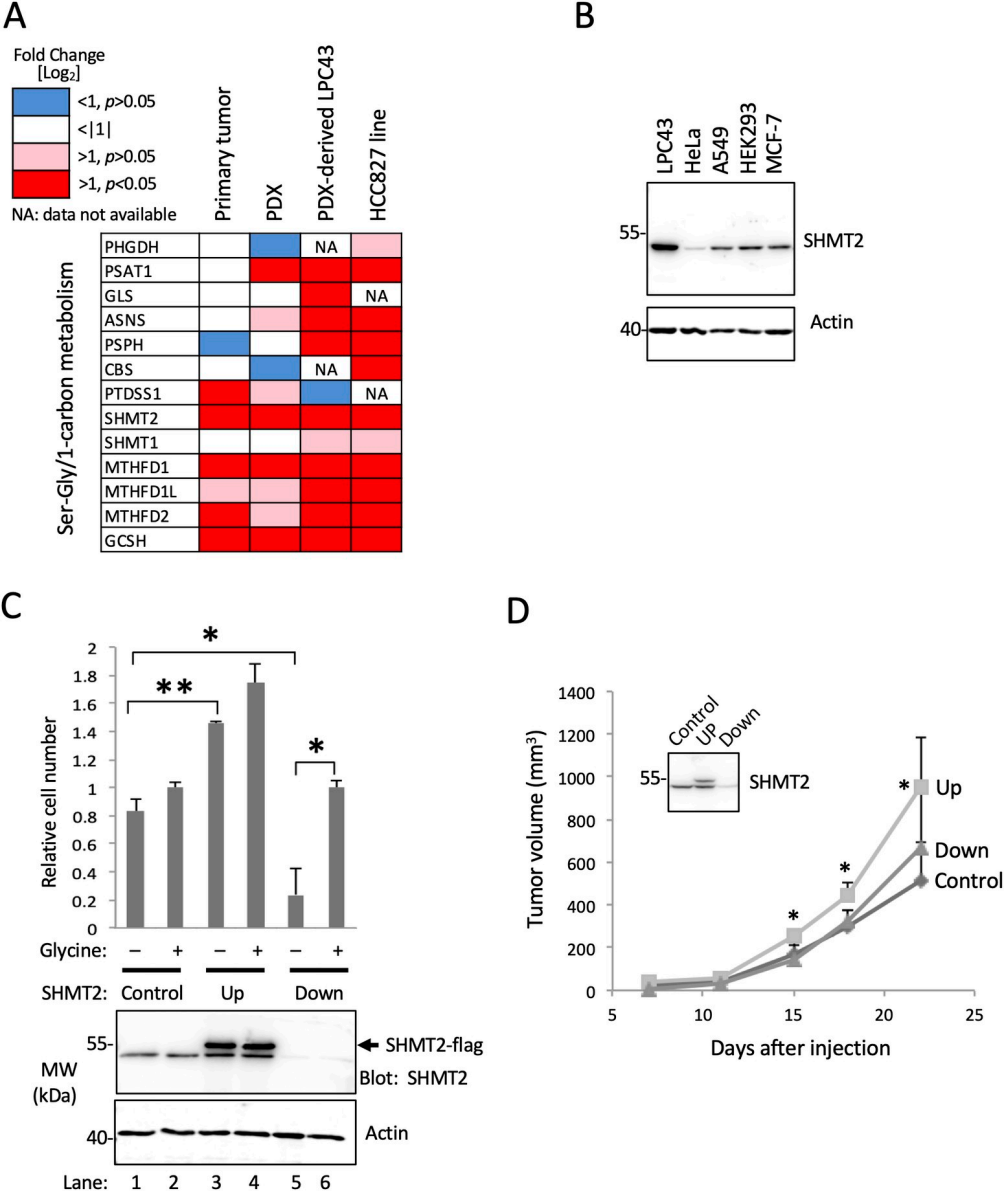
Expression of one carbon metabolism proteins in a lung tumor and derived models and effect of modulated SHMT2 expression. (A) Comparison of expression of SHMT2 and proteins involved in one-carbon metabolism in indicated tissue and cell lines. Protein intensities were analyzed by MS and quantified by using MaxQuant software. Protein expression in the four indicated samples were normalized with a control protein (HIST2H3A) and then compared to levels measured in normal lung tissue; (B) Endogenous SHMT2 expression in five indicated cell lines. Western blotting was carried out with the indicated antibodies. (C) Histogram showing effect of SHMT2 protein expression on HeLa cell proliferation. HeLa-Ss cells were induced for ectopic expression of SHMT2-Flag (Up) or shRNA-mediated knockdown of endogenous SHMT2 (Down). Cells were tested in synthetic medium lacking glycine and containing dialyzed (glycine-free) serum, and supplemented without (–) or with (+) 100 mM glycine. The aligned anti-SHMT2 western blot (lower panel) shows protein expression of ectopic and endogenous SHMT2 (as indicated), and expression actin as an indication of equal protein loading. (D) Effect of SHMT2 expression on tumor growth *in vivo*. SCID mice bearing HeLa-Ss cells with induced expression of SHMT2-Flag (UP) or shRNA-SHMT2 (Down) were monitored for xenograft tumor growth. Mean tumor volumes (± SD) for each group (n = 4 or 5) are shown. The inserted box is a representative western blot of three tumor samples confirming changes in SHMT2 protein levels as indicated.

A previous multi-omic analysis of aggressive, early-stage NSCLC revealed metabolism proteome signatures highly recapitulated between patient-matched primary and patient-derived xenograft (PDX) tumors [9]. Relative to a patient-matched normal lung controls, among the more than 800 metabolism and metabolite transport proteins measured in tumors, the enzyme SHMT2 was the most highly upregulated, at 14-fold in primary tumors, and 22-fold in PDX tumors [9]. Here we analyzed expression of SHMT2, and 12 additional enzymes involved in serine/glycine/1-carbon metabolism in a NSCLC primary tumor, a cognate PDX model, cell line LPC43 that was derived from the PDX, and the established lung-adenocarcinoma-derived cell line HCC827 (Fig 1A, S2 Table in S1 Data). Relative to normal lung controls, SHMT2 along with MTHFD1 and GCSH were significantly upregulated (p <0.05) in all of the transformed tissue/cell samples. These three enzymes plus PSAT1 were significantly upregulated in the PDX and both cell lines, while an additional six enzymes were significantly upregulated in one or both of the cell lines (Fig 1A, S2 Table in S1 Data). These results suggest an increased capacity relative to normal lung for serine/glycine/1-carbon metabolism in primary lung tumors and derived models.

In order to test the requirement for SHMT2 protein expression on cell proliferation, we sought to modulate its expression in a model cell line. Western blotting for SHMT2 in a set of established cell lines (LPC43, HeLa, A549, HEK392, and MCF-7) showed that NSCLC-derived LPC43 has relatively high endogenous SHMT2 expression, whereas SHMT2 protein is relatively low in HeLa (Fig 1B). In their survey of gene essentiality in five representative cell lines, Moffat and co-workers observed SHMT2 to be required by HeLa cells [38]. Therefore, HeLa were deemed likely to be SHMT2 dependent and chosen for further analysis of SHMT2 function. A HeLa derivative named HeLa-Ss was engineered to express Flag epitope-tagged SHMT2 in response to Tet treatment, and to have diminished SHMT2 protein expression in response to IPTG-induced expression of an SHMT2-directed shRNA. In HeLa-Ss ectopic SHMT2 expression was evident within hours of Tet treatment, whereas decreased SHMT2 was observed after three days of IPTG treatment (Fig 1C, S1A Fig). In HeLa and LPC43 backgrounds, ectopically expressed SHMT2-Flag appeared to co-localize with a mitochondrial marker similar to endogenous SHMT2 (S1B Fig). In control experiments, the Tet and IPTG treatments used to modulate SHMT2 expression in HeLa-Ss cells did not affect proliferation or colony formation in the parental HeLa-Trex cells (S1C Fig).

Since SHMT2 catalyzes the production of glycine, the requirement for glycine as a growth supplement was examined as a function of SHMT2 expression. In glycine-free medium HeLa-Ss proliferation was SHMT2-dependent; enhanced by ectopic SHMT2 over-expression (Fig 1C, compare lanes 1 and 3); and strongly impaired by SHMT2 knock down (Fig 1C, lane 5). Proliferation of SHMT2 knock down cells was restored by addition of glycine to growth medium (Fig 1C, lane 6). Therefore, in the absence of SHMT2, HeLa cells are auxotrophic for glycine. As shown in Fig 1C, in cells expressing endogenous SHMT2 (Control; lanes 1, 2), or following induction of ectopic over-expression (Up; lanes 3, 4), addition of glycine to the growth medium slightly but not statistically significantly increased cell proliferation (lanes 1–4), which is a trend consistent with the known stimulatory effect of glycine on transformed cell proliferation [6]. The impaired proliferation of SHMT2 knockdown HeLa-Ss cells in glycine-free medium (Fig 1C, lane 5) was not likely due to apoptosis, since we found no evidence for cleaved PARP protein compared with positive controls (methotrexate, transforming growth factor alpha, sodium orthovanadate) that caused PARP cleavage (S1D Fig).

As a further examination of the effects of SHMT2 on cell transformation, soft agar colony formation was measured. HeLa-Ss with or without over-expression of SHMT2 formed comparable numbers of colonies with or without supplementation of glycine in the growth medium (S2 Fig). However, in glycine-free medium, but not in medium with added glycine, colony formation was decreased by SHMT2 knockdown (S2 Fig). Colony formation by the parental cells was not affected by the Tet or IPTG treatments (S2 Fig).

Next we tested if the lung cancer patient-derived-xenograft-derived cell line LPC43, which expresses a much higher level of endogenous SHMT2 compared with HeLa (see Fig 1B) was sensitive to glycine deprivation as a function of SHMT2 expression. A cell variant with inducible knockdown of SHMT2 was generated (LPC43-shSHMT2), and IPTG-induced knockdown of SHMT2 protein expression was verified (S3 Fig). In these cells, knockdown of SHMT2 protein expression inhibited cell proliferation in glycine-depleted medium, but only under hypoxia/low oxygen conditions (S3B Fig).

In order to test the effects SHMT2 expression on tumor formation *in vivo*, HeLa-Ss cells were implanted in immune deficient mice (see Materials and Methods). Induced over-expression of SHMT2-Flag (Up) promoted a statistically significant increase in HeLa-Ss xenograft tumor growth whereas knockdown of SHMT2 (Down) had no effect (Fig 1D). Modulation of SHMT2 expression in xenograft tumors of mice treated with doxycycline (SHMT2-Up) and IPTG (SHMT2-Down) was confirmed by western blot (Fig 1D, insert).

### Identification of SHMT2 associated proteins

**Fig 2:**
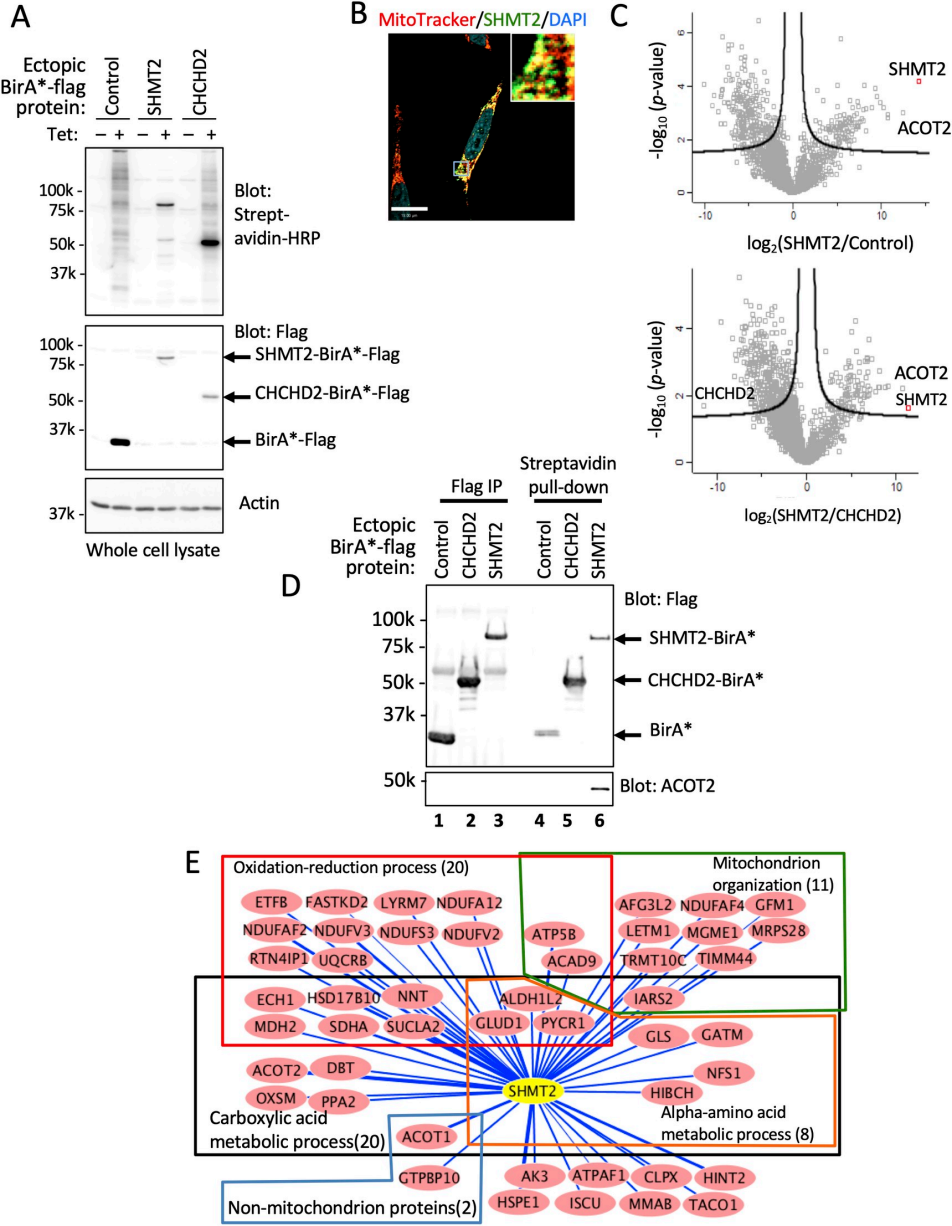
Analysis of SHMT2 interactome by proximity-dependent biotinylation identification (BioID). (A) Western blot of whole cell lysates from HeLa cells expressing Tet-induced proteins as indicated; proteins have both C-terminal Flag and BirA* tags. (B) Colocalization of MitoTracker Red probe and SHMT2-BirA*-Flag protein. (C) Volcano plot of significance versus fold-change on the y and x axes, respectively, comparing biotinylated proteins identified in SHMT2-BirA-expressing cells and that in two BirA*controls (n = 3). Proteins outside of the lines are significantly different between the indicated two samples. (D) Western blot validation of SHMT2 interacting proteins. Tagged SHMT2 and co-immunoprecipitated partner, ACOT2, was detected with antibodies to SHMT2 and ACOT2. (E) Network analysis of SHMT2 interactome by Cytoscape. Forty-eight proteins significantly different (>32-fold) between ectopic SHMT2 and controls were selected. Proteins and their interactions are shown as nodes and edges. Edges indicate the amount of biotinylated protein in SHMT2-BirA-Flag samples calculated as log_10_ LFQ signal/protein molecular weight. Gene ontology consortium (http://geneontology.org/) was used for functional classification.

Towards understanding mechanisms of SHMT2 effects on cell proliferation and tumor growth, we sought to identify proteins associated with or proximal to SHMT2 by using proximity biotinylation (BioID) methodology [19]. HeLa, which is a well-established platform for interactome studies was adopted for these experiments [24,39]. Expression and subcellular localization of SHMT2-BirA*-Flag protein were observed by Western blot (Fig 2A) and immunostaining (Fig 2B). Fluorescence microscopy analysis suggested co-localization of ectopic SHMT2 with mitochondria (Fig 2B, S4 Fig). Biotinylated proteins identified from HeLa cells expressing SHMT2-BirA*-Flag were compared with controls expressing BirA*-Flag alone and a previously described, non-SHMT2-interacting mitochondrial fusion protein CHCHD2-BirA*-Flag [25] (Fig 2C). ACOT2, an SHMT2 interacting protein identified by BioID (Fig 2C) was confirmed by western blot (Fig 2D). Forty-nine proteins (including SHMT2 itself) were significantly differentially biotinylated (>32-fold; p <0.05) in cells expressing SHMT2-BirA*-Flag compared with cells expressing BirA*-Flag and CHCHD2-BirA*-Flag (Table 1 and S3 Table in S1 Data). According to gene ontology (GO) annotation (http://pantherdb.org/tools/), 47 out of 49 SHMT2-associated proteins identified by BioID were mitochondrial proteins. Among the interacting proteins PANTHER classified oxidoreductase as one of the top enriched groups, and with 20 proteins involved in oxidation-reduction processes and as members of respiration complex 1 or 3 (Fig 2E, S4 Table in S1 Data). We note that another complex 1 protein NDUFS6 showed significant association with SHMT2 (>100-fold over control BirA*, p <0.05; see S3 Table in S1 Data) but since this interaction was only some 30-fold greater than its association with the other control protein CHCHD2-BirA*, it failed the stringent requirements for inclusion in Table 1. Additional proteins that showed statistically significant association with SHMT2 compared with one or other control are listed in S3 Table in S1 Data.

**Table 1:** Proteins with significantly high biotinylation in cells expressing SHMT2-BirA.

Gene	Protein	*Amount Log2 [SHMT2/Control)	SHMT2 Association Rank
SHMT2	Serine hydroxymethyltransferase, mitochondrial	14.24	
ACOT2	Acyl-coenzyme A thioesterase 2, mitochondrial	12.42	1
ACOT1	Acyl-coenzyme A thioesterase 1	10.73	2
HSPE1	10 kDa heat shock protein, mitochondrial	10.71	3
NDUFV3	NADH dehydrogenase [ubiquinone] flavoprotein 3, mitochondrial	10.26	4
LETM1	LETM1 and EF-hand domain-containing protein 1, mitochondrial	9.90	5
NDUFAF2	Mimitin, mitochondrial	9.45	6
NNT	NAD(P) transhydrogenase, mitochondrial	9.29	7
AFG3L2	AFG3-like protein 2	8.95	8
MDH2	Malate dehydrogenase, mitochondrial;Malate dehydrogenase	8.27	9
TACO1	Translational activator of cytochrome c oxidase 1	8.26	10
IARS2	Isoleucine—tRNA ligase, mitochondrial	8.05	11
HINT2	Histidine triad nucleotide-binding protein 2, mitochondrial	8.00	12
PYCR1	Pyrroline-5-carboxylate reductase 1, mitochondrial	8.00	13
NFS1	Cysteine desulfurase, mitochondrial	7.61	14
GTPBP10	GTP-binding protein 10	7.40	15
HSD17B10	3-hydroxyacyl-CoA dehydrogenase type-2	7.39	16
SUCLA2	Succinyl-CoA ligase [ADP-forming] subunit beta, mitochondrial	7.36	17
ECH1	Delta(3,5)-Delta(2,4)-dienoyl-CoA isomerase, mitochondrial	7.28	18
ALDH1L2	Mitochondrial 10-formyltetrahydrofolate dehydrogenase	7.25	19
GLS	Glutaminase kidney isoform, mitochondrial	7.22	20
GATM	Glycine amidinotransferase, mitochondrial	7.21	21
SDHA	Succinate dehydrogenase [ubiquinone] flavoprotein subunit, mitochondrial	7.14	22
TRMT10C	Mitochondrial ribonuclease P protein 1	6.94	23
ACAD9	Acyl-CoA dehydrogenase family member 9, mitochondrial	6.92	24
NDUFS3	NADH dehydrogenase [ubiquinone] iron-sulfur protein 3, mitochondrial	6.83	25
GLUD1	Glutamate dehydrogenase, mitochondrial	6.75	26
ETFB	Electron transfer flavoprotein subunit beta	6.72	27
TIMM44	Mitochondrial import inner membrane translocase subunit TIM44	6.63	28
ISCU	Iron-sulfur cluster assembly enzyme ISCU, mitochondrial	6.62	29
UQCRB	Cytochrome b-c1 complex subunit 7	6.61	30
PPA2	Inorganic pyrophosphatase 2, mitochondrial	6.52	31
NDUFAF4	NADH dehydrogenase [ubiquinone] 1 alpha subcomplex assembly factor 4	6.47	32
HIBCH	3-hydroxyisobutyryl-CoA hydrolase, mitochondrial	6.44	33
CLPX	ATP-dependent Clp protease ATP-binding subunit clpX-like, mitochondrial	6.37	34
NDUFA12	NADH dehydrogenase [ubiquinone] 1 alpha subcomplex subunit 12	6.34	35
NDUFV2	NADH dehydrogenase [ubiquinone] flavoprotein 2, mitochondrial	6.27	36
GFM1	Elongation factor G, mitochondrial	6.25	37
AK3	GTP:AMP phosphotransferase AK3, mitochondrial	6.20	38
FASTKD2	FAST kinase domain-containing protein 2	6.13	39
OXSM	3-oxoacyl-[acyl-carrier-protein] synthase, mitochondrial	6.08	40
ATP5B	ATP synthase subunit beta, mitochondrial	5.93	41
MRPS28	28S ribosomal protein S28, mitochondrial	5.87	42
RTN4IP1	Reticulon-4-interacting protein 1, mitochondrial	5.81	43
MMAB	Cob(I)yrinic acid a,c-diamide adenosyltransferase, mitochondrial	5.71	44
LYRM7	Complex III assembly factor LYRM7	5.62	45
MGME1	Mitochondrial genome maintenance exonuclease 1	5.55	46
ATPAF1	ATP synthase mitochondrial F1 complex assembly factor 1	5.27	47
DBT	Lipoamide acyltransferase component of branched-chain alpha-keto acid dehydrogenase complex, mitochondrial	5.20	48

*Proteins with >32 fold increased biotinylation in SHMT2-BirA cells compared with control (Cont)-BirA and CHCHD2-BirA cells (n = 3, p<0.05)

In another set of experiments AP-MS was used to assess proteins physically associated with Flag-tagged SHTM2. This analysis revealed associated proteins corresponding to a cytosolic BRISC complex (S5 Table in S1 Data, S5 Fig) in agreement with Zheng *et al*. [2] and suggesting that in these experiments ectopic SHMT2-Flag was cytosolic as expected for the SHMT2α isoform [2]. BRISC complex proteins were not identified as SHMT2-associated by the BioID method.

### Effects of SHMT2 expression on the proteome

**Fig 3:**
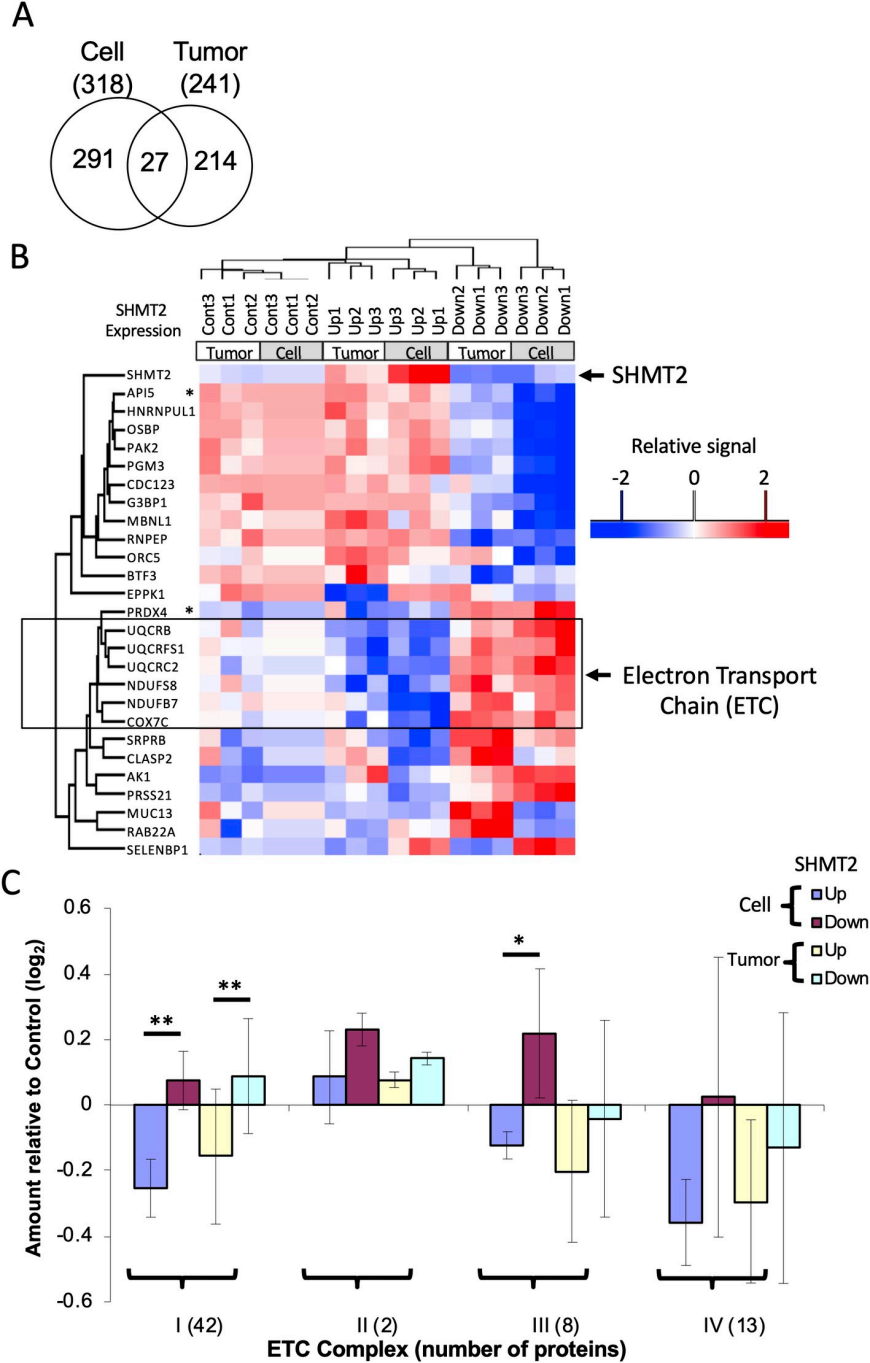
SHMT2 expression levels influence the expression of electron transport chain proteins. Proteins from HeLa-Ss cells and HeLa-Ss tumors with different SHMT2 expression levels (Control, Up, and Down) were analyzed by MS by using a TMT protocol. (A) Venn analysis of proteins differentially expressed as a function of SHMT2 in HeLa-Ss cells versus tumors. Proteins with significantly differential expression among control (Cont), SHMT2-Up, and SHMT2-Down was determined by ANOVA analysis. (B) Unsupervised hierarchical cluster analysis of 27 proteins significantly differentially expressed as a function of SHMT2 level in both cells and tumors (as depicted in A, above). ETC: electron transport chain. * PRDX4: Peroxiredoxin-4; API5: Apoptosis inhibitor 5. (C) Relative quantification of electron transport chain proteins of complex I, II, III and IV. * *p* < 0.01; ** *p* <0.001 (Student’s t-test).

To further understand mechanisms of SHMT2 effects on cell proliferation and metabolism, we measured the proteome and a set of metabolites as a function of SHMT2 expression in HeLa-Ss cells. Tandem mass tag (TMT) methodology was used to differentially label with isobaric mass tags proteins extracted from HeLa-Ss cells grown *in vitro* and *in vivo*. Three biological replicates each of control, SHMT2 over-expression, and SHMT2 knockdown for a total of 9 samples were prepared from *in vitro* and *in vivo* samples. Each group of 9 samples was labeled along with one channel of the 10-plex set as a reference mixture comprising an equal amount of each of the 18 samples (i.e. from cultured cells and xenograft tumors). In aggregate, a total of 8772 proteins were quantified as MS^3^ TMT reporter signals (S6 Table in S1 Data). Compared with control, the average SHMT2 expression was 15-fold and 2.6-fold higher in SHMT2 over-expressing cells (UP) cells and xenograft tumors, respectively. SHMT2 expression was 1.6-fold and 2.6-fold lower in SHMT2 knock-down cells (Down) and xenograft tumors, respectively (S6 Table in S1 Data, S6 Fig). These finding were consistent with western blot analyses as shown in Fig 1. ANOVA analysis identified 318 significantly changed proteins (Benjamini and Hochberg adjusted *p* value <0.05, S7 Table in S1 Data) among three groups of HeLa-Ss cells grown in culture (Control, SHMT2-Up and SHMT2-Down) and 241 proteins (*p*<0.05, S8 Table in S1 Data) among three groups of HeLa-Ss xenograft tumors. Twenty-seven proteins were identified as significantly changed as a function of SHMT2 expression in both cells and tumors (Fig 3A, S9 Table in S1 Data). Unsupervised clustering of samples based on the abundance of the 27 proteins divided the 18 samples into 3 groups according to SHMT2 expression (i.e. Control, Up, Down; Fig 3B) and further separated each of these sets into 2 subgroups corresponding to cell samples and tumor samples. The 27 proteins separated into two major clusters, with one having an expression pattern concordant with SHMT2 (upper cluster in Fig 3B), and another in which protein expression was opposite to that of SHMT2. In this latter set, 6 of 14 proteins are mitochondrial electron transport chain (ETC) components (outlined in Fig 3B). Gene set enrichment analysis showed that proteins involved in electron transport were significantly enriched among the 318 proteins differentially expressed as a function of SHMT2 expression in HeLa-Ss cells and the 241 proteins differentially expressed as a function of SHMT2 expression *in vivo* (S10 Table in S1 Data). We further examined all identified ETC proteins in cells and in tumors and found that complex 1 proteins were significantly reduced in SHMT2 over-expressing cells and tumors and conversely were slightly elevated in SHMT2 knock-down cells and tumors (Fig 3C, S11 Table in S1 Data, *p*<0.001). Other proteins that appeared to change as a function of SHMT2 expression were peroxiredoxin-4 (PRDX4) and apoptosis inhibitor 5 (API5), two proteins involved in cell apoptosis (see asterisks, Fig 3B). Proteins involved in Ser/Gly/1-carbon metabolism as listed in Fig 1 trended towards increased expression that was not statistically significant in SHMT2-Up cells and tumors (S6 Fig).

### Changes in proliferation and/or metabolites as a function of SHMT2 expression and sodium benzoate treatment *in vitro* and *in vivo*

**Fig 4:**
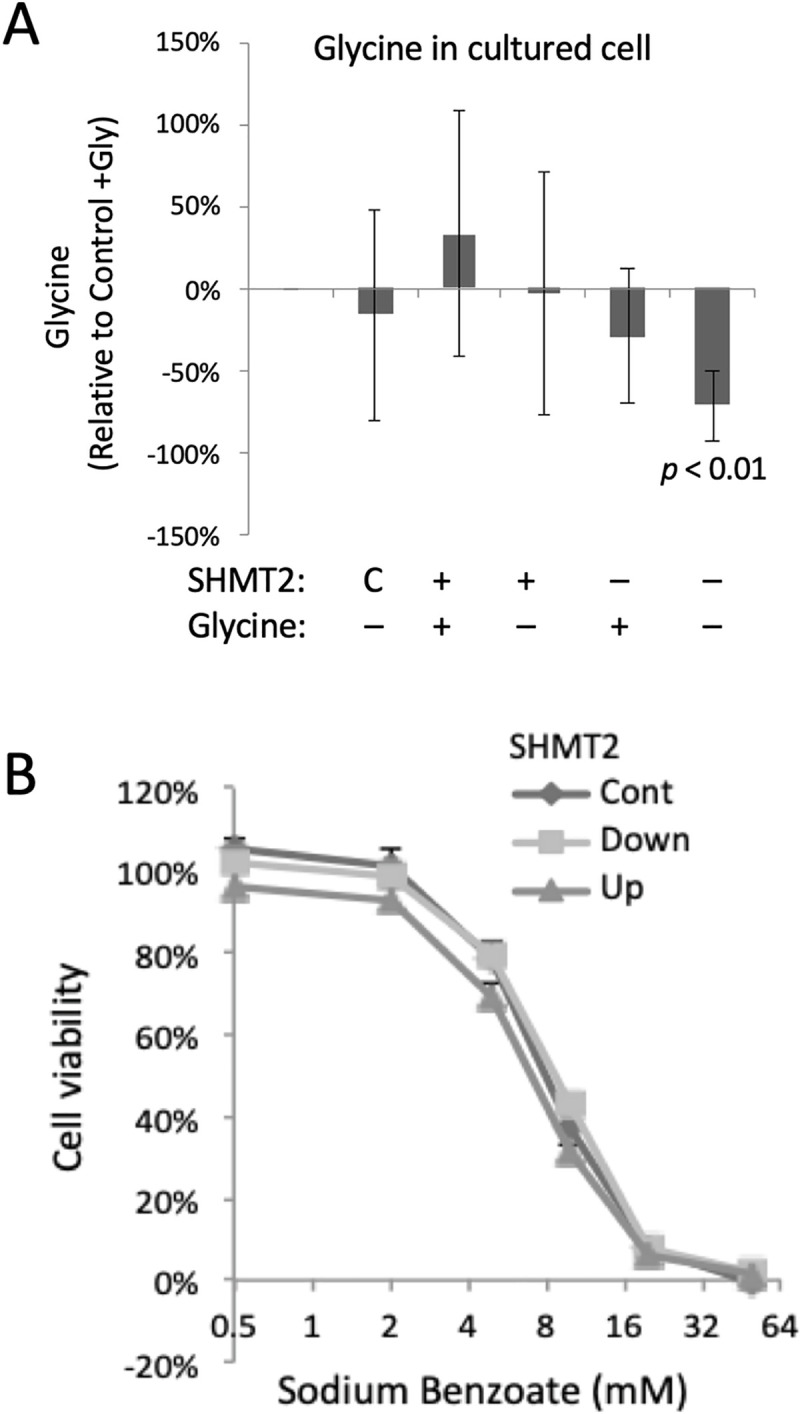
Analysis of glycine and proliferation *in vitro* as a function of SHMT2 and sodium benzoate. HeLa-Ss cells were grown in normal media under SHMT2 Tet induction (Up) or IPTG knock-down (Down) treatment for 72 h. Intracellular metabolites were extracted and analyzed by selected reaction monitoring mass spectrometry, SRM, n = 5 to 8 biological replicates. (A) In HeLa-Ss cells relative levels of intracellular glycine levels were measured as a function of endogenous SHMT2 expression (control; C) or elevated (+) or decreased (–) expression of SHTM2 (described above), and without (–) or with (+) glycine supplementation in growth medium. (B) HeLa-Ss cell viability as a function of sodium benzoate concentration as indicated in uninduced cells (control, cont) or following induction of ectopic SHMT2 (Up) or knockdown of SHMT2 expression (Down).

Targeted metabolite profiling was carried out on 89 metabolites by SRM (metabolites and their transitions are listed in S1 Table in S1 Data). Targeted measurement of glycine is shown as an example of metabolite quantification (S7 Fig). Except for glycine and serine, HeLa-Ss cells grown in culture had similar levels of metabolites when grown in medium with and without glycine supplementation and with or without SHMT2 modulation (S12 Table in S1 Data). Serine levels were 1.5-times higher in SHMT2 knockdown cells compared with SHMT2 overexpressing cells (p = 0.048). Conversely, glycine levels were were increased 1.8-fold in SHMT2 overexpressing cells compared with SHMT2 knockdown cells (p = 0.053) (S12 Table in S1 Data). In medium lacking added glycine, cellular glycine levels were significantly decreased by almost 70% in HeLa SHMT2 Down cells (Fig 4A; S7 Fig, S13 Table in S1 Data).

SHMT2 knock down impaired cell proliferation of cells *in vitro* only when deprived of an extracellular source of glycine (Fig 1C column 6) but did not slow xenograft tumor growth (Fig 1D). We hypothesized the apparent continued proliferation of SHMT2 knock down tumor cells was due to the availability of extracellular glycine in the mouse, and that a further systemic reduction of glycine might reveal an SHMT2 dependence and thereby impair tumor growth. Benzoate is a treatment for (nonketotic) hyperglycinemia because it results in the conversion of glycine to hippurate in mitochondria and thereby reduces glycine levels systemically [40]. Sodium benzoate treatment of cultured HeLa-Ss reduced cell proliferation in a dose-dependent manner (Fig 4B; IC50 11.8 ±1.8 mM, n = 3), but was independent of cellular SHMT2 expression level, and was associated with apoptosis as indicated by increased amounts of cleaved PARP in benzoate-treated cells (S8 Fig). The most highly elevated metabolite after SHMT2 knock down with or without benzoate treatment *in vitro* was phosphoribosylaminoimidazolecarboxamide (AICAR), an intermediate in the *de novo* purine nucleotide synthesis pathway that was increased 7-fold in SHMT2 knockdown cells with or without glycine supplementation (p<0.05; S9 Fig). We speculate AICAR accumulated because of an inability to convert it to phosphoribosylformylaminoimidazolecarboxamide (FAICAR), which requires as a co-factor 10-formyl-THF that is derived from the SHMT2 product methylene-THF [3].

**Fig 5:**
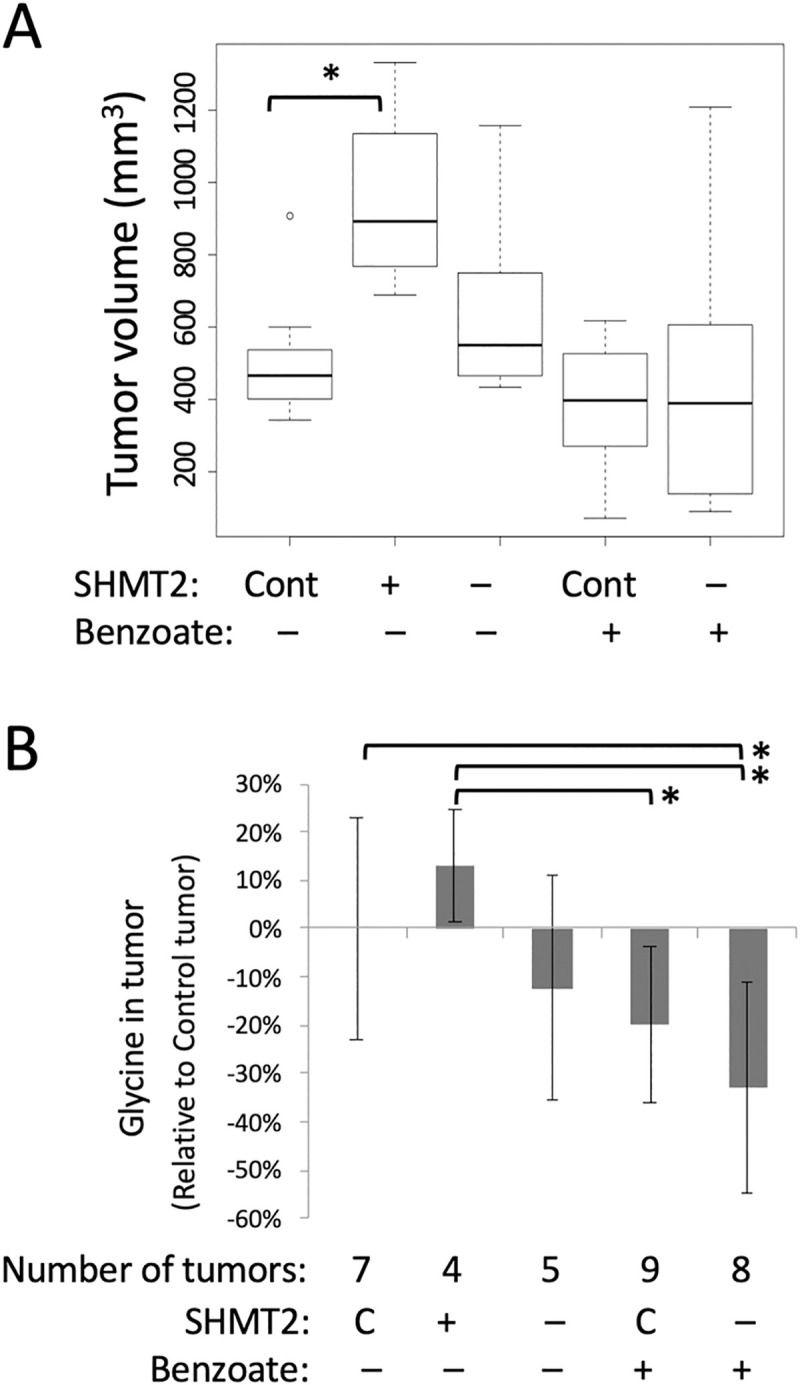
Effect of sodium benzoate on HeLa-Ss cell growth *in vivo*. (A) Effect of benzoate on tumor growth. HeLa-Ss xenograft tumor growth in SCID mice was monitored as a function of endogenous SHMT2 expression (C, control), ectopic over-expression (+), or knockdown (–), and without (–) or with (+) sodium benzoate treatment. Tumor volumes were measured 21 days after cell implantation and shown as boxplots for each group of mice (n = 4 to 9). Median tumor volume is shown as a line inside the box, and first and third quartiles were shown as bottom and top of box. (B) Glycine levels were measured by SRM and relative changes in tumor samples are shown. Abbreviations are Control, C; Ectopic over-expression (+) or knockdown (–) of SHMT2 and without (–) or with sodium benzoate (+) as indicated.* *p*<0.05.

Modulation of SHMT2 protein in tumors by ectopic expression and knockdown was confirmed by western blot (S10A Fig). Ectopic over-expression of SHTM2 in xenograft tumors was associated with a significant increase in tumor volume (Fig 5A), whereas we did not measure a significant change in tumor growth after SHMT2 knockdown (Fig 5A, compare lanes 1 and 3). Benzoate treatment of mice did not significantly reduce HeLa-Ss tumor size (Fig 5A, S10B Fig). Mice were given 1% sodium benzoate in drinking water, which is an amount less than the established toxicity limit of 2% for mice [23]. The average benzoate consumption for mice was approximately 1000 mg/kg/day, which is comparable to the dose of 750 mg/kg/day that is used for the treatment of pediatric nonketotic hyperglycinemia [40].

Metabolites that changed as a function of SHMT2 modulation or benzoate treatment *in vivo* included glycine, serine, and hippurate (S11 Fig). Relative to control mice, in benzoate treated SHMT2 knockdown mice glycine was significantly down (p = 0.013) and hippurate was significantly increased (p = 0.030) (S14 Table in S1 Data). Compared with control tumors, SHMT2 over-expression *in vivo* increased glycine levels by 13%, and SHMT2 knock down decreased glycine by 12% (Fig 5B, S15 Table in S1 Data). Benzoate treatment *in vivo* reduced glycine by 20% and in combination with SHMT2 knockdown further reduced glycine by 33% (S15 Table in S1 Data and S11A Fig). Therefore, the most pronounced reduction in relative glycine levels (70%) was seen in HeLa-Ss SHTM2-knockdown cells grown *in vitro* without glycine and treated with benzoate (Fig 4B).

### Other functional analyses on SHMT2-Ss cells

**Fig 6:**
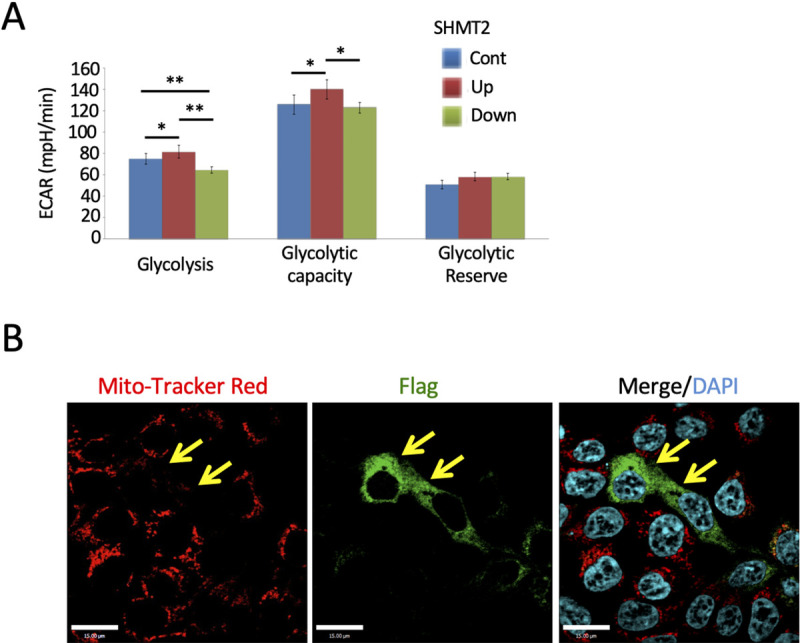
Changes of SHMT2 expressions affected glycolysis and reduced capacity of mitochondria in HeLa-Ss cells. (A) Seahorse XFe96 Extracellular Flux analyzer was used to measure extracellular acidification rate (ECAR) as an indicator of glycolysis. Basal, maximum and reserved ECAR rate among control, SHMT2 over-expression and knockdown HeLa-Ss cells was compared. All data are presented as mean ± SD from 3 independent experiments with 6–14 replicate wells per experiment. * *p* < 0.05 and ** *p* < 0.001 (Student T-test). (B) Reduced MitoTracker Red level in SHMT2 over-expressing cells. Forty-eight hours after transfection with the SHMT2-Flag vector, HeLa cells were incubated with MitoTracker Red and fixed and stained with anti-Flag antibodies (green). Yellow arrows indicate SHMT2-Flag-expressing cells. Scale bars are 15 μm.

Given the observations that SHMT2 interacted with multiple mitochondrial electron transport chain components (Fig 2E) and affected their expression (Fig 3C), mitochondria oxygen consumption rates (OCR) were analyzed as a measure of mitochondrial function. HeLa-Ss cells with different SHMT2 expression levels showed similar oxygen consumption rate signatures as a function of the indicated stress conditions (S12A Fig). Extracellular acidification rate (ECAR) was taken as a measure of glycolysis. SHMT2 over-expression led to a small significant increase in ECAR upon addition of glucose (i.e. basal glycolysis) and oligomycin (i.e. maximum glycolysis) (Fig 6A and S12B Fig). SHMT2 knock-down had the opposite trend, suggesting that SHMT2 over-expressing cells were more glycolytic under basal conditions and had higher glycolytic capacity.

Immunofluorescence microscopic analysis showed that transient over-expression of SHMT2 in HeLa cells inhibited fluorescence of the reagent MitoTracker Red CMTMRos, which requires oxidation in mitochondria (Fig 6B). Quantitative measurement of MitoTracker Red CMTMRos in HeLa-Ss cells (S13 Fig) and biochemical analysis of cellular glutathione levels by using a mono-chlorobimane (MCB) assay (S13 Fig) indicated a significant increase in glutathione and state of reduction in cells expressing ectopic SHMT2, in agreement with previous publications [4,7].

Western blot analysis of two mitochondrion proteins (PGC-1α and TFam) and PCR quantification of mitochondrion DNA copy number indicate no detectable changes in mitochondrial biogenesis and mtDNA copy number after alteration of SHMT2 expression (S14 Fig).

## Discussion

SHMT2 is highly upregulated at the protein level in NSCLC and participates in several aspects of metabolism critical for transformed cell proliferation and survival, including the production of methylene-tetrahydrofolate (THF) and glycine, and including through coupled reactions 10-formyl-THF and NADPH. Protein-protein interactions are known to occur among enzymes involved in folate-dependent metabolism [41] and represent a basic mechanism of protein regulation [42]. We therefore sought to further explore the function of SHMT2 by examination of its protein-protein interactions by using the complementary approaches of AP-MS and BioID. AP-MS has been applied to characterize physically associated proteins that co-immunoprecipitate [39], whereas BioID may additionally identify proteins that are less stably associated and/or within a roughly 10-nm-proximity [24,43]. Both approaches revealed interacting proteins, but without overlap. Lack of congruence between AP-MS and BioID has been observed by comparison of chromatin-associated interacting proteins by AP-MS and BioID [44]. The SHMT2-BirA* fusion protein co-localized with mitochondria and biotinylated proteins identified by MS were predicted mitochondrial proteins. By contrast AP-MS analysis of SHMT2-Flag identified only the cytosolic BRISC complex in agreement with Zheng and Greenberg [2]. This finding suggests that the SHMT2-Flag protein was localized outside mitochondria similar to the SHMT2α isoform [1,45]. Further structural analysis would be required to better understand the apparent cytosolic localization of SHMT2-Flag.

The tandem mass tag (TMT) approach is a powerful method to characterize and compare proteomes. By using this method, we observed a significant reverse correlation between SHMT2 and a group of mitochondrial electron transport chain (ETC) complex 1 and 3 proteins (Fig 3). However, the reduction in expression of ETC proteins in SHMT2 over-expression cells was not accompanied by changes in mitochondrial oxygen consumption rates (Fig 6). Serine catabolism by SHMT2 has been shown to affect complex I protein expression by epigenetic regulation of DNA methylation [14] and mitochondrial tRNA [15,16]. These studies used inhibitors [13] or SHMT2 knock out methods [14,15,17] to impair SHMT2 function in cells. Our data showed that over-expression of SHMT2, a phenomenon seen in tumors, reduced ETC protein expression. Due to high electron flux through individual complexes, the ETC is the major source of mitochondrial reactive oxygen species (ROS). Complex I and complex III have been identified as prime superoxide-generating sites [46,47]. While the precise mechanism of ROS generation by these complexes is not fully understood [48], we speculate that lowered expression of complex 1 and complex 3 associated with elevated SHMT2 expression may reduce ROS production and possibly enhance cell survival under hypoxic conditions. Our BioID result showed interactions between SHMT2 and ETC complex 1 and 3 proteins (Fig 2), which suggests that SHMT2 may affect the stability of these protein complexes through protein-protein interactions.

Over-expression of SHMT2 also regulated the expression of other proteins (Fig 3B). Over-expression of SHMT2 up-regulated API5 (apoptosis inhibitor 5) and down-regulated PRDX4 (peroxiredoxin-4). API5 overexpression was shown to be associated with tumor progression and poor prognosis in patients with cervical cancer [49]. Weak PRDX4 expression in lung adenocarcinoma had a significantly close relationship with pathologically poor differentiation, highly invasive characteristics, and recurrence [50]. Therefore, we speculate these additional aspects of the cancer phenotype may be linked to SHMT2.

SRM measurement of metabolites is confounded by different ionization efficiencies of pure, standard compounds and metabolite sample mixtures [28]. Therefore, relative SRM peak areas of metabolites were compared among samples. This revealed expected changes in serine and glycine as a function of SHTM2 expression level, and an apparent accumulation of AICAR upon SHMT2 knockdown in vitro and in xenograft tumors. We speculate AICAR accumulates because its conversion to FAICAR requires formyl-THF derived from the SHMT2 product methylene-THF [3].

SHMT2 knockdown rendered the cultured HeLa cell system auxotrophic for glycine confirming a context-specific critical role of this enzyme. This observation is consistent with those of Jain et al. (2012) that various rapidly dividing transformed cells are dependent on SHMT2-dependent glycine production and may display glycine auxotrophy upon SHMT2 knockdown, and with Puck and co-workers (Kao et al. 1969) who characterized glycine-dependent CHO cell mutants that were impaired for measured SHMT activity. However, while we achieved inducible SHMT2 knockdown *in vivo*, this did not prevent tumor growth. We hypothesized that xenograft tumors continued to grow with reduced SHMT2 because they were not starved for glycine. We tested this notion by using sodium benzoate, which is used clinically to reduce systemic glycine by conversion to hippurate [40,51]. We observed hippurate production and potentiation of SHTM2-associated glycine depletion after sodium benzoate treatment *in vitro* and *in vivo*. However, benzoate-induced inhibition of proliferation *in vitro* was independent of SHMT2 modulation, but accompanied by apoptosis. Reduced SHMT2 protein expression and associated glycine reduction, even when effectively potentiated by benzoate treatment was insufficient to inhibit HeLa xenograft tumor growth.

In conclusion, the genetic, proteomic, and metabolomic results presented in this study offer new insights into the function and mechanism of action of SHMT2 upregulation in cancer. In the context of the HeLa-Ss inducible system, induced ectopic over-expression of SHMT2 was sufficient to increase tumor cell proliferation *in vitro* and *in vivo*. Analysis of protein-protein interactions showed SHMT2 was found associated with various proteins involved in mitochondrial respiration, and to affect their expression level. The work reinforces the notion that SHMT2 protein levels affect levels of metabolites associated with serine/glycine and folate/1-carbon pathways, including NADPH, and glutathione. Furthermore, our findings suggest that SHMT2 ‘s role in metabolism may be coupled through protein interactions with mitochondrial functions involving respiration complexes 1 and 3. Lastly, the surprising interaction of SHMT2 with ACOT2 suggests that further research is warranted to explore if SHMT2 function(s) are linked via this interaction to the regulation of acyl-CoAs, free fatty acids, and CoASH.

## Supporting information

S1 File (PDF)

S1 Data (XLSX)

S1 Fig SHMT2 cellular localization and inducible SHMT2 expression in HeLa-Ss cells. (A) WB analysis of whole cell lysates (WCL) from HeLa-Ss cells with Tet or IPTG treatments. HeLa-Ss cells were engineered for inducible ectopic expression of SHMT2-Flag (Up) and shRNA against SHMT2, which knocked down endogenous SHMT2 expression (Down). Results shown are representative of three independent experiments. (B) Immunofluorescence imaging of LPC43 cells transfected with SHMT2-GFP vector and HeLa-Ss cell with Tet treatment. After 48 h of SHMT2-GFP transfection, LPC43 cells were incubated with MitoTracker Red for 30 min in cell culture incubator and fixed. After 24 h Tet induction, HeLa-Ss cells were incubated with MitoTracker Red and fixed, which were then stained with anti-SHMT2 antibodies (green) and DAPI (blue for nuclear staining). Yellow color indicates co-localization. Scale bars are 15 μm. (C) Effect of Ted and IPTG on parent HeLa cell growth. HeLa cells were treated with Tet or IPTG for 3 d and cell number was measured by a crystal violet method and compared to cells grown in glycine supplemented medium. (D) Effect of SHMT2 expression and glycine depletion on apoptosis. Cleaved PARP is used as indicator of apoptosis and WB was used to analyze WCL from HeLa-Ss cells with indicated treatment.
(PDF)

S2 Fig Soft agar assay on SHMT2 cell growth. Soft agar assay was used to assess effect of target gene on anchorage independent growth. The conditions on cell culture were marked beside each well. Total numbers of colonies for a single representative experiment are shown in bar graphs below.
(PDF)

S3 Fig Effect of reduced SHMT2 expression on the growth of LPC43, a lung tumor cell line. (A) Effect of IPTG inducible shRNA against SHMT2 in LPC43 cell. LPC43 is a human cell line derived from a NSCLC patient-derived xenograft (25). (B) LPC43 cell growth under normoxia and hypoxia conditions. LPC43 cells were treated with IPTG for 7 days and split on the same time as control cells, 3 d later the cell number was measured by a crystal violet method. Cells were tested in synthetic medium lacking glycine, and supplemented with dialyzed (glycine-free) serum (-Gly) or with addition of 100 mM glycine (+Gly). SHMT2 Down cells were treated with IPTG, therefore, had reduced SHMT2 expression. Control (Cont) cells were LPC43 cell without IPTG treatment.
(PDF)

S4 Fig BirA protein localization in BioID assay. (A and B) Immunofluorescence imaging of SHMT2-BirA (A) and Cont-BirA (B) alone in HeLa cells. DNA encoding Flag-tagged BirA or SHMT2-BirA were stably incorporated into HeLa-Trex genomic DNA, and Ted treatment of cells induced their expression. After 24 h Tet induction, HeLa-SHMT2-BirA-Flag or HeLa-Cont-BirA-Flag cells were incubated with MitoTracker Red and fixed and then stained with anti-SHMT2 antibodies or anti-Flag antibodies (green). Yellow color indicates co-localization. Scale bars are 15 μm.
(PDF)

S5 Fig SHMT2 expression in HEK-293 cell and AP-MS analysis of SHMT2 associated proteins. (A) SHMT2-GFP transient over-expression in HEK-293 cells. After 48 h of SHMT2-GFP transfection, HEK-293 cells were incubated with MitoTracker Red for 30 min in cell culture incubator and fixed. Yellow color indicates co-localization of mitochondrion maker (Red) and SHMT2 (Green). (B) Changes of SHMT2 expression in HEK-293 engineered cells. shRNA against SHMT2 was introduced into HEK-293 cell by lentivirus with puromycin selection; ectopic over-expression of Flag-tagged SHMT2 was introduced into HEK cells by pcDNA3 vector with G418 selection. WB analysis of WCL on stable cell lines was shown. (C) Volcano plot analysis of anti-Flag immunoprecipitation of SHMT2 over-expression cells from three biological repeats for each sample. Red dots indicate SHMT2 specifically associated proteins and * indicates proteins in BRISC complex.
(PDF)

S6 Fig Changes of proteins involved in serine/Glycine/1-carbon synthesis in HeLa-Ss cells and tumors. Functions of proteins are illustrated above the heatmap. Heatmap is the relative amount of proteins compared with control samples. Proteins with pink color (>1) indicates a relative abundance more than control; blue (<1) indicates less.
(PDF)

S7 Fig Quantification of glycine by SRM. (A) A chromatograph of glycine standard measured by SRM and quantified by Skyline software. (B) Dose-response curve of a glycine standard measured with SRM. (C) Quantification of glycine in indicated samples. Left two panels are chromatographs of glycine from sample and standard. Right top panel is the alignment of retention time of samples and standards. Right bottom panel is the quantification result of indicated samples and standard.
(PDF)

S8 Fig Treatment with sodium benzoate induced apoptosis of HeLa cells. WB was carried out with indicated antibodies. Cleaved PARP is used as an indicator of cell apoptosis.
(PDF)

S9 Fig Measurement of AICAR by SRM in cultured cells. Targeted metabolite, AICAR, was extracted from HeLa-Ss cell *in vitro* and measured by SRM. Data were analyzed as described in Fig 5; * *p* < 0.05.
(PDF)

S10 Fig Benzoate effect on HeLa-Ss tumor growth *in vivo*. (A) SHMT2 expression in ten indicated tumor samples. Western blot was carried out with indicated antibody. (B) Effect of benzoate treatment on tumor growth in vivo. SCID mice bearing HeLa-Ss cell lines treated with Benz plus or minus shRNA-SHMT2 (Down), were monitored for tumor growth. The mean tumor volumes (± SD) for each group (n = 4 to 9) are shown.
(PDF)

S11 Fig Effect of sodium benzoate on metabolites of tumor cells grown *in vivo*. (A-E) Targeted metabolites were extracted from tumor and measured by SRM. Data were analyzed as described in Fig 5; * *p* < 0.05.
(PDF)

S12 Fig Changes in SHMT2 expression affect glycolysis, but not mitochondrial respiration in HeLa-Ss cells. A Seahorse XFe96 Extracellular Flux analyzer was used to measure mitochondrial oxygen consumption rate (OCR) and extracellular acidification rate (ECAR). (A) Real time OCR of control (blue), SHMT2 over-expression (red) and knockdown (green) HeLa-Ss cells in mitochondrial stress tests. (B) Real time ECAR measurements of control, SHMT2 over-expression and knockdown HeLa-Ss cells in glycolysis stress tests. All data were presented as the mean ± standard deviation from 6–14 replicate wells per experiment. * *p* < 0.05 and ** *p* < 0.001 (Student’s t-test).
(PDF)

S13 Fig Reduced MitoTracker Red signal and reduced glutathione levels in SHMT2 over-expressing HeLa cells. (A) Quantification of MitoTracker Red amount in indicated cells. HeLa-Ss cells were cultured in 96 cell plates, labeled with MitoTracker Red, and measured at 579/612 nm. (B) Relative amount of glutathione in living cells. Glutathione was measured with monochlorobimane (MCB) assay. * *p*<0.05 compared with control sample (Cont).
(PDF)

S14 Fig Measurement of mitochondrial proteins and DNA copy number. (A) WB analysis of two mitochondrial proteins (PGC-1α and TFam) with indicated antibodies. (B) Quantification of mitochondrion DNA copy number. Total DNA was extracted from indicated cells. Quantitative PCR with specific primers for entire D-loop of mtDNA DNA and β-actin were used to quantify DNA amounts as described previously [52]; relative amount are shown.
(PDF)

## Abbreviations

AP-MS: affinity purification mass spectrometry
IPTG: isopropyl β-D-1-thiogalactopyranoside
LC-MS/MS: liquid chromatography-tandem mass spectrometry
NSCLC: non-small cell lung carcinoma
SHMT2: serine hydroxymethyltransferase 2
