# Supplementary material for: Cancer proteome and metabolite changes linked to SHMT2
Source: PLoS One. 2020 Sep 9;15(9):e0237981. doi: 10.1371/journal.pone.0237981 (PMC7480864; doi:10.1371/journal.pone.0237981)
Supplement: S1 File — (PDF) [file pone.0237981.s001.pdf]

Supporting information for Blots

Protocol for western blot analysis

For western blotting analysis, cells on plates were washed in cold PBS twice and lysed in NP40 lysis buffer (50 mM Tris/HCl pH7.5, 100 mM NaCl, 1% NP40, 0.1% sodium deoxcholate, 10% glycerol, 1mM EDTA, 100 mM NaF, 1 mM sodium orthovanadate, protease inhibitor tablets-EDTA-free (Thermo Fisher Scientific)) and rocked for 30 min at 4°C.

For western blotting analysis of xenograft tissues, a fragment of xenograft tumors was chopped to pieces. NP40 lysis buffer was used (roughly 200 μL to 10 mg of tissues) to lyse tissues for 1 hour at 4°C.

Insoluble cell or tissue debris was removed by centrifuging at 18000 g for 10 min at 4 °C and protein amounts were measured by using Pierce BCA Protein Assay Kit (Thermo Fisher Scientific). Lysates were diluted to 2 mg protein/ml and added equal volume of 2xSDS Laemmli sample buffer.

Total cell or tissue lysates (10 μg protein/10 μl sample/lane) were separated by SDS-PAGE (routinely 15 well gel) and electro-transferred onto nitrocellulose membranes (Thermo Fisher Scientific). The membrane then was blocked in 1× TBS buffer (Bio Basic) and 0.1% Tween-20 (TBST buffer) with 5% (weight / volume) non-fat milk (Bio-Rad). Primary antibodies (1:1000 to 1:5000 dilution) were added to TBST buffer with 1% (weight / volume) non-fat milk and the membrane was incubated at 4 °C overnight. After washing with TBST buffer, the secondary antibody was added at 1:5000 to 1:10000 dilution and incubated for 1 hour at room temperature. Anti-rabbit or anti-mouse IgG 2nd antibodies with HRP conjugate or with DyLight™ 800 4xPEG conjugate or 680 conjugate were purchased from Cell Signaling Tech. After three to four washes, Luminata Forte Western HRP substrate was used for HRP conjugate 2nd antibody blot. Chemiluminescence or fluorescence on membrane was recorded by IS (Image Studio) program in Odyssey Fc imaging instrument (LI-COR) according to manufacturer's instruction, and imaging files were export as .tif or .png format with 300 dpi resolution for record.

Notes:

1. Nitrocellulose membrane with protein transferring was cut into small pieces or stripes according to the detection of targeted proteins. This practice will reduce the consumption of 1st and 2nd antibodies and uneven background. For example, the molecular weight of Actin is 42 kDa, so blot around 40 kDa protein marker band was cut for anti-Actin blot.
2. Blue protein standard bands (Thermo or Bio-Rad products) were recorded at 700 nm fluorescence channel and overlaid on chemiluminescence channel or 800 nm fluorescence channel blot.
3. Regions outside of blot in original .tif file were routinely cropped to reduce the size of files for achieve by Adobe Photoshop® program.
4. All relevant samples in a figure result were run on the same gel, and any adjustments to improve visibility of the data were applied to the entire image.

Fig 1B

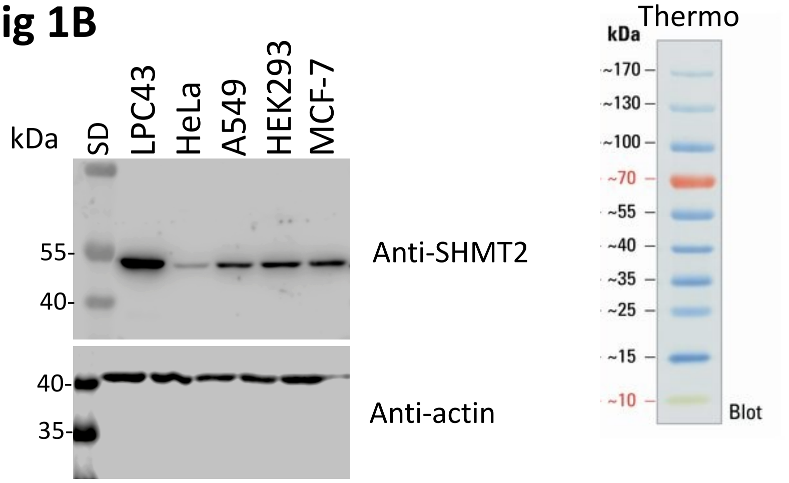

Fig 1C

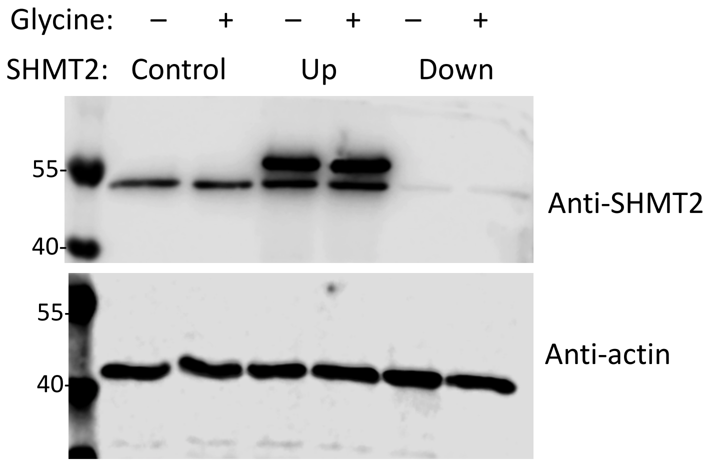

Fig 1D insert

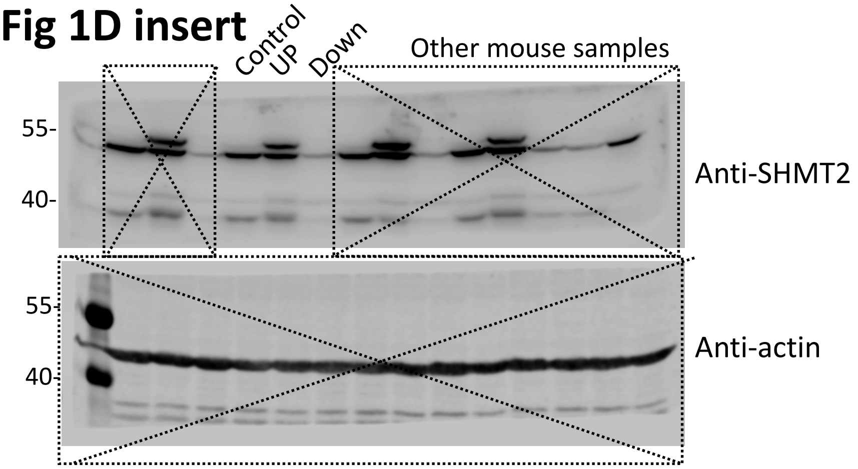

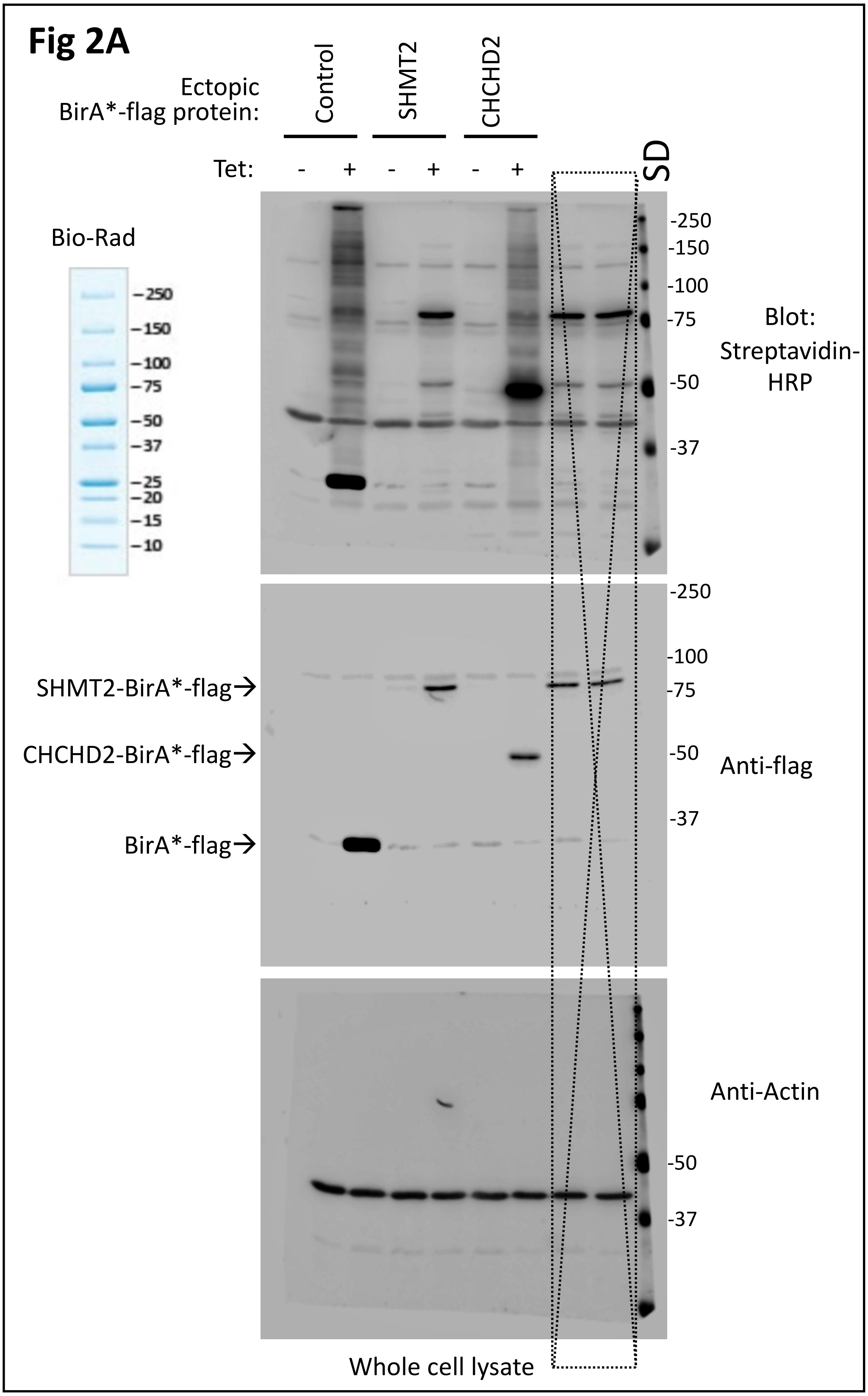
Fig 2A
Ectopic BirA*-flag protein:
Control
SHMT2
CHCHD2
Tet:
- + - + - +
SD
Bio-Rad
250
150
100
75
50
37
25
20
15
10
Blot: Streptavidin-HRP
Anti-flag
Anti-Actin
SHMT2-BirA*-flag
CHCHD2-BirA*-flag
BirA*-flag
Whole cell lysate

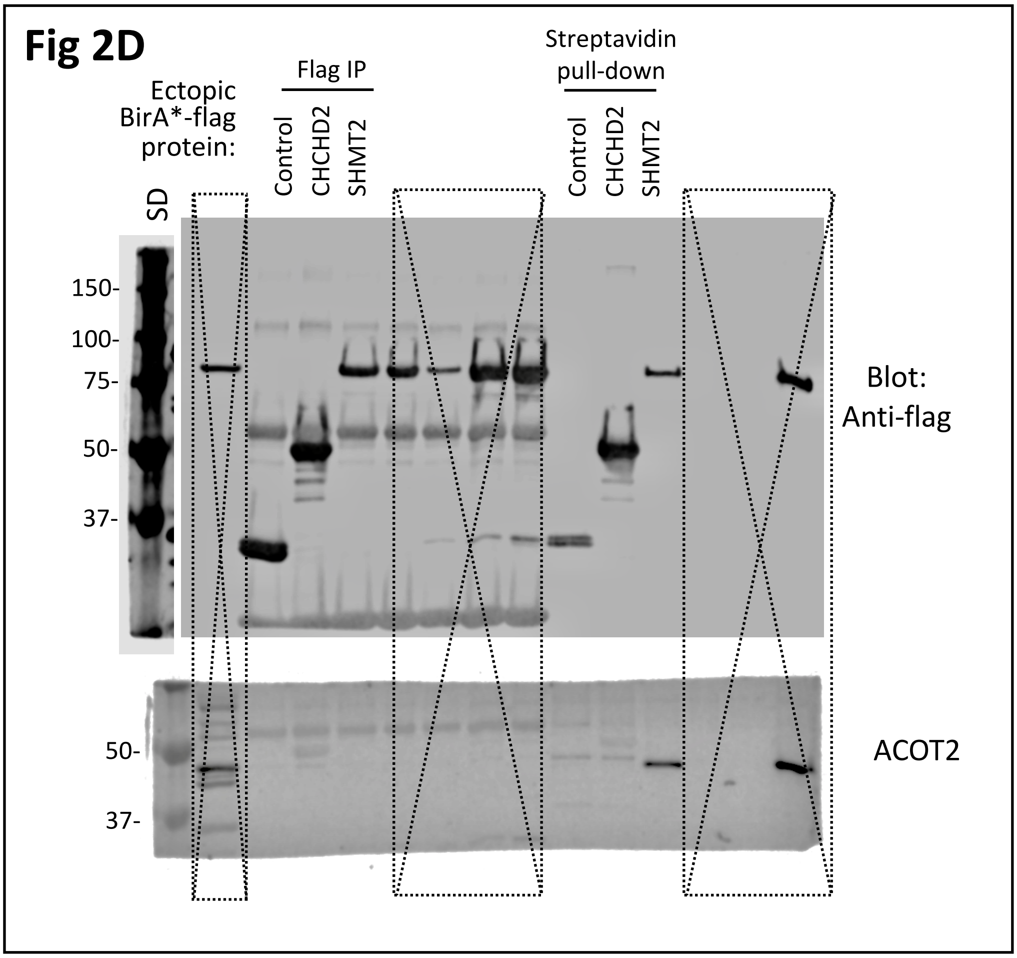
Fig 2D
Ectopic BirA*-flag protein:
Flag IP
Streptavidin pull-down
SD
Control
CHCHD2
SHMT2
Control
CHCHD2
SHMT2
150-
100-
75-
50-
37-
Blot: Anti-flag
50-
37-
ACOT2

S1A Fig

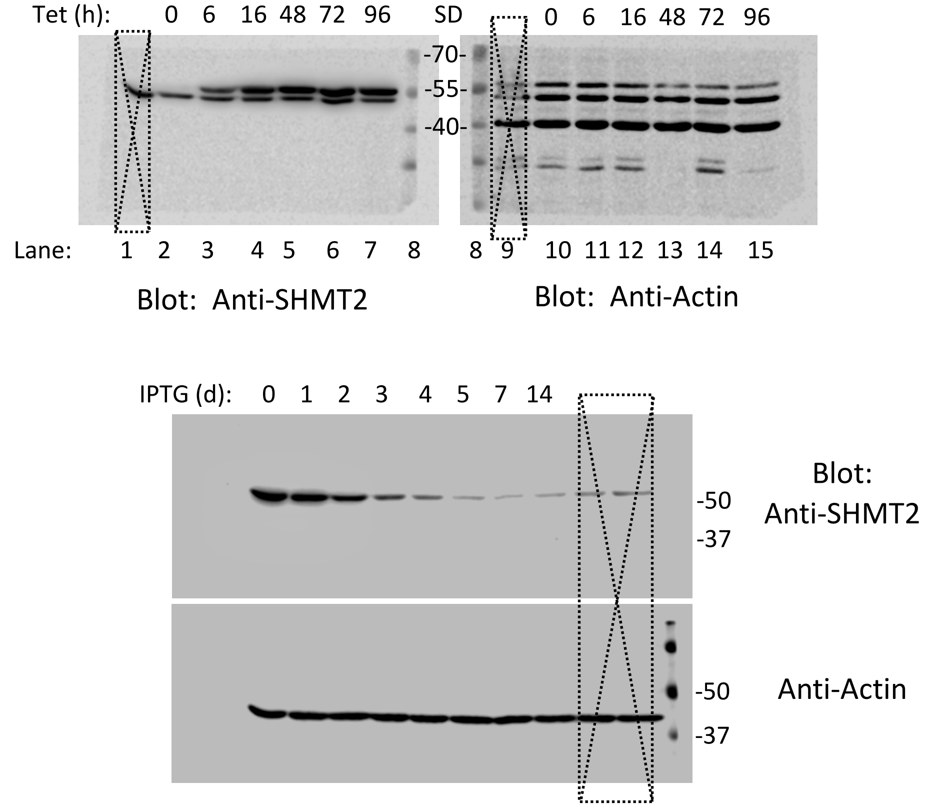

S1D Fig

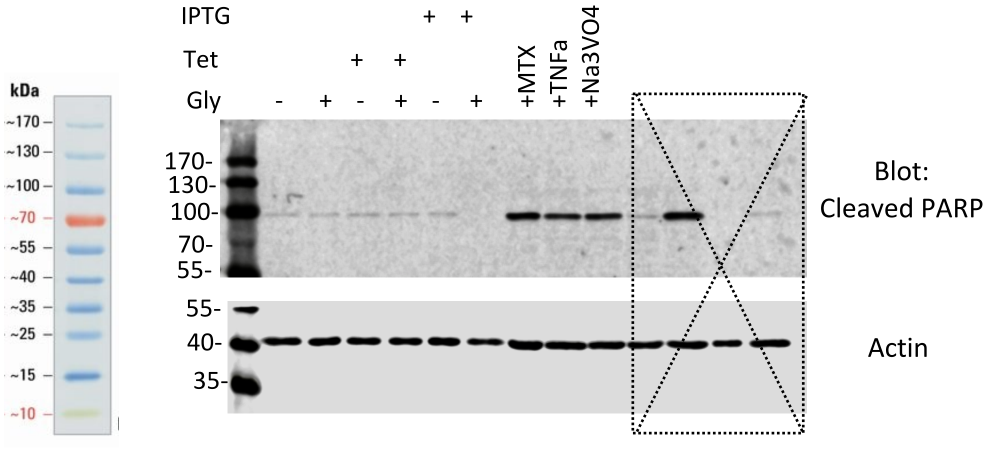

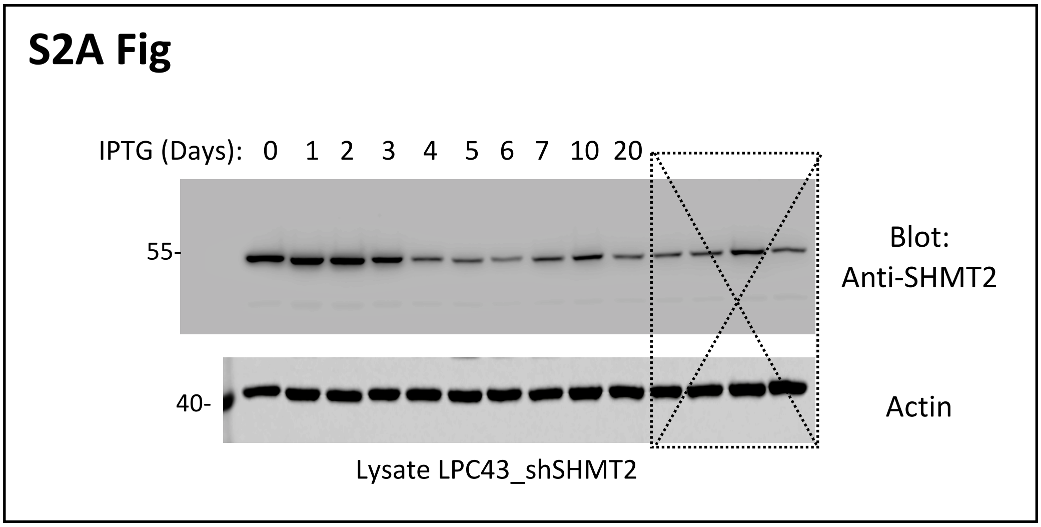
S2A Fig
IPTG (Days): 0 1 2 3 4 5 6 7 10 20
55-
40-
Blot:
Anti-SHMT2
Actin
Lysate LPC43_shSHMT2

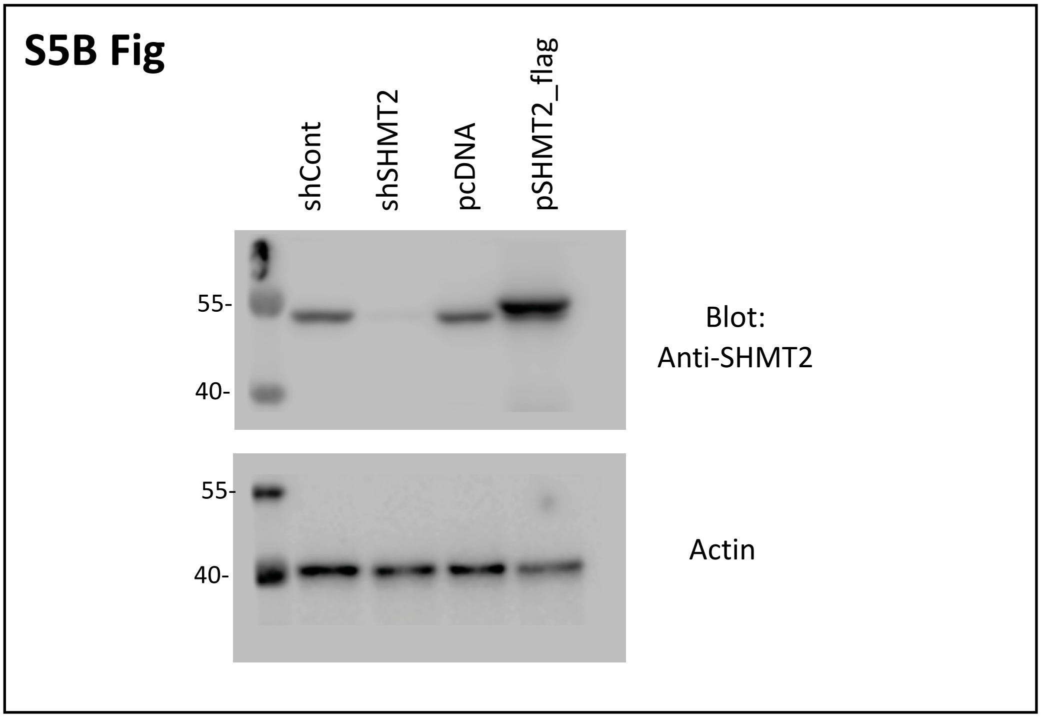
S5B Fig
shCont
shSHMT2
pcDNA
pSHMT2_flag
55-
40-
Blot:
Anti-SHMT2
55-
40-
Actin

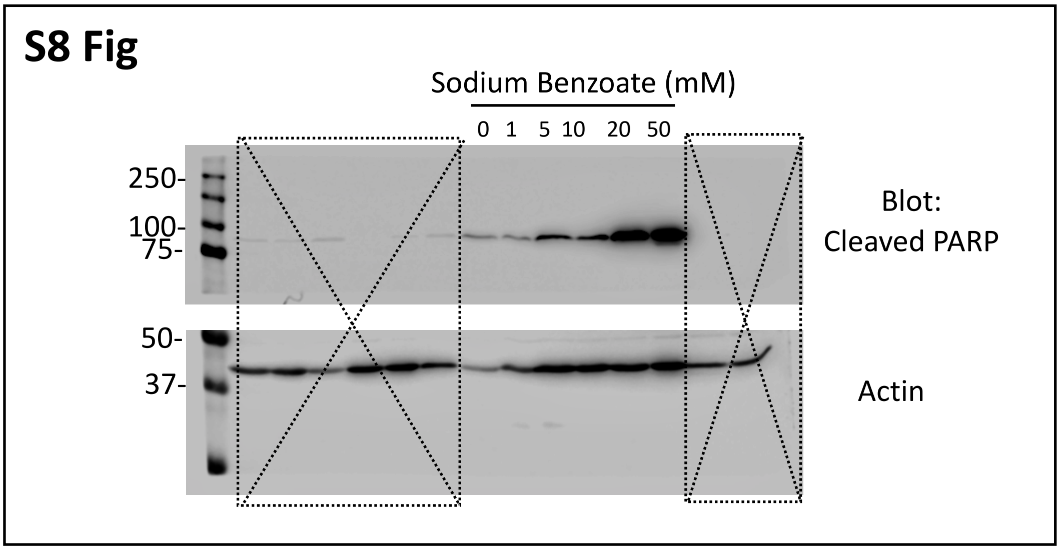
S8 Fig
Sodium Benzoate (mM)
0 1 5 10 20 50
250-
100-
75-
Blot:
Cleaved PARP
50-
37-
Actin

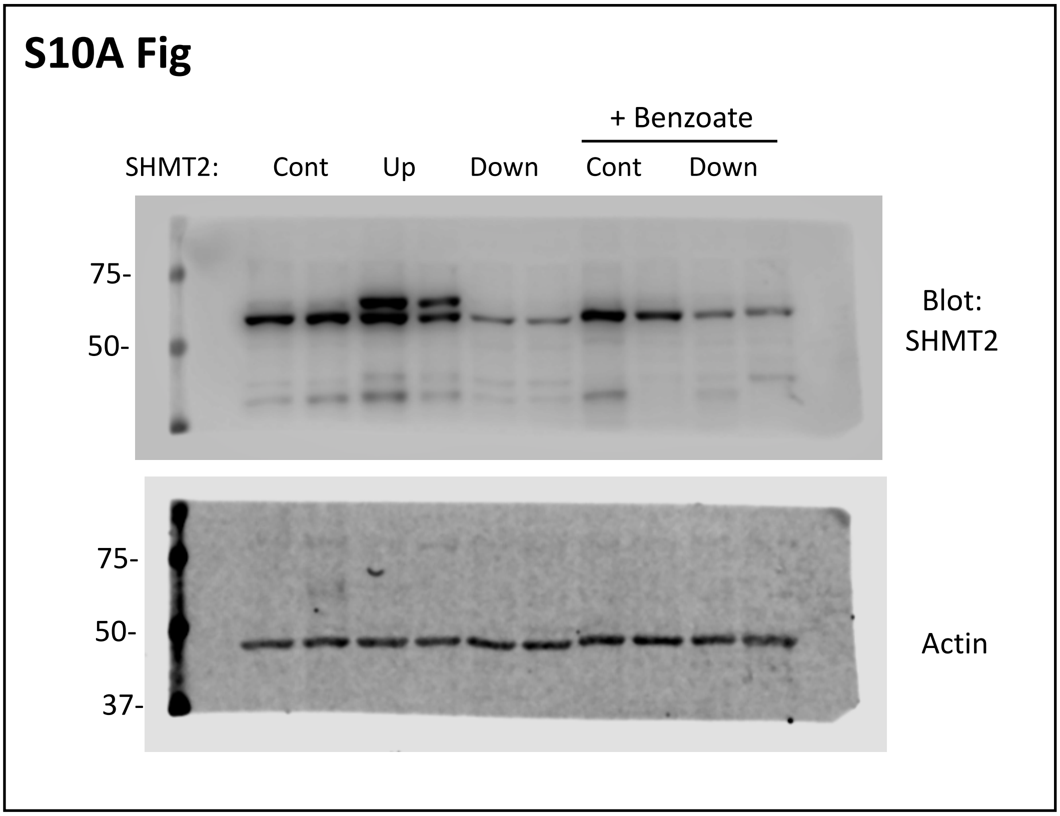
S10A Fig
+ Benzoate
SHMT2:
Cont
Up
Down
Cont
Down
75-
50-
Blot:
SHMT2
75-
50-
37-
Actin

S14 Fig

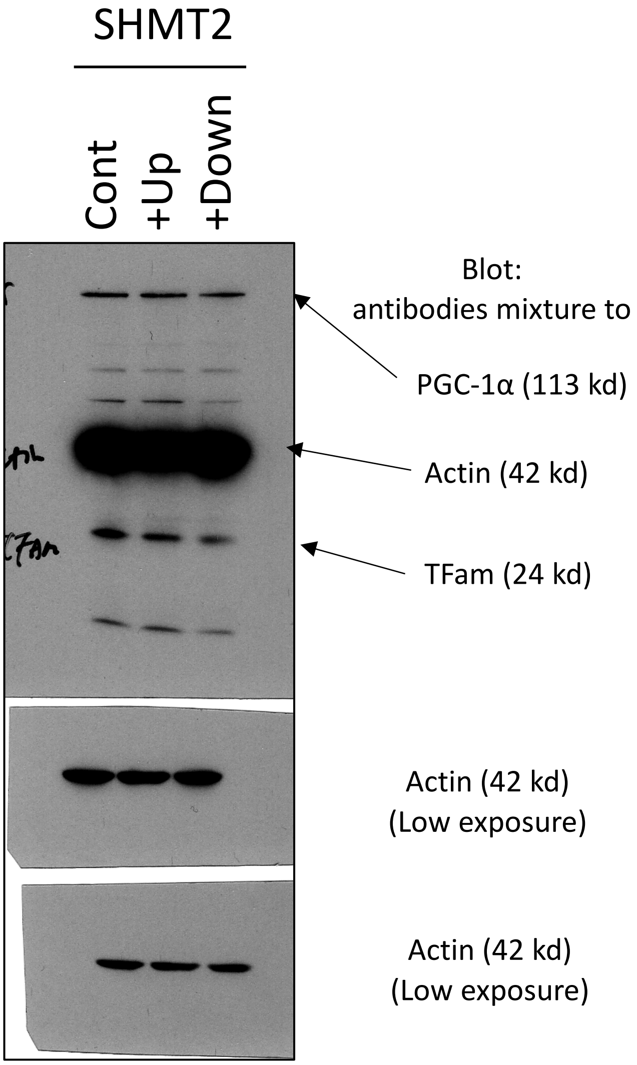

Note: This western blot was developed on film.
