# Supplementary figures and images for: Cancer proteome and metabolite changes linked to SHMT2
Source: PLoS One. 2020 Sep 9;15(9):e0237981. doi: 10.1371/journal.pone.0237981 (PMC7480864; doi:10.1371/journal.pone.0237981)

S1 Fig

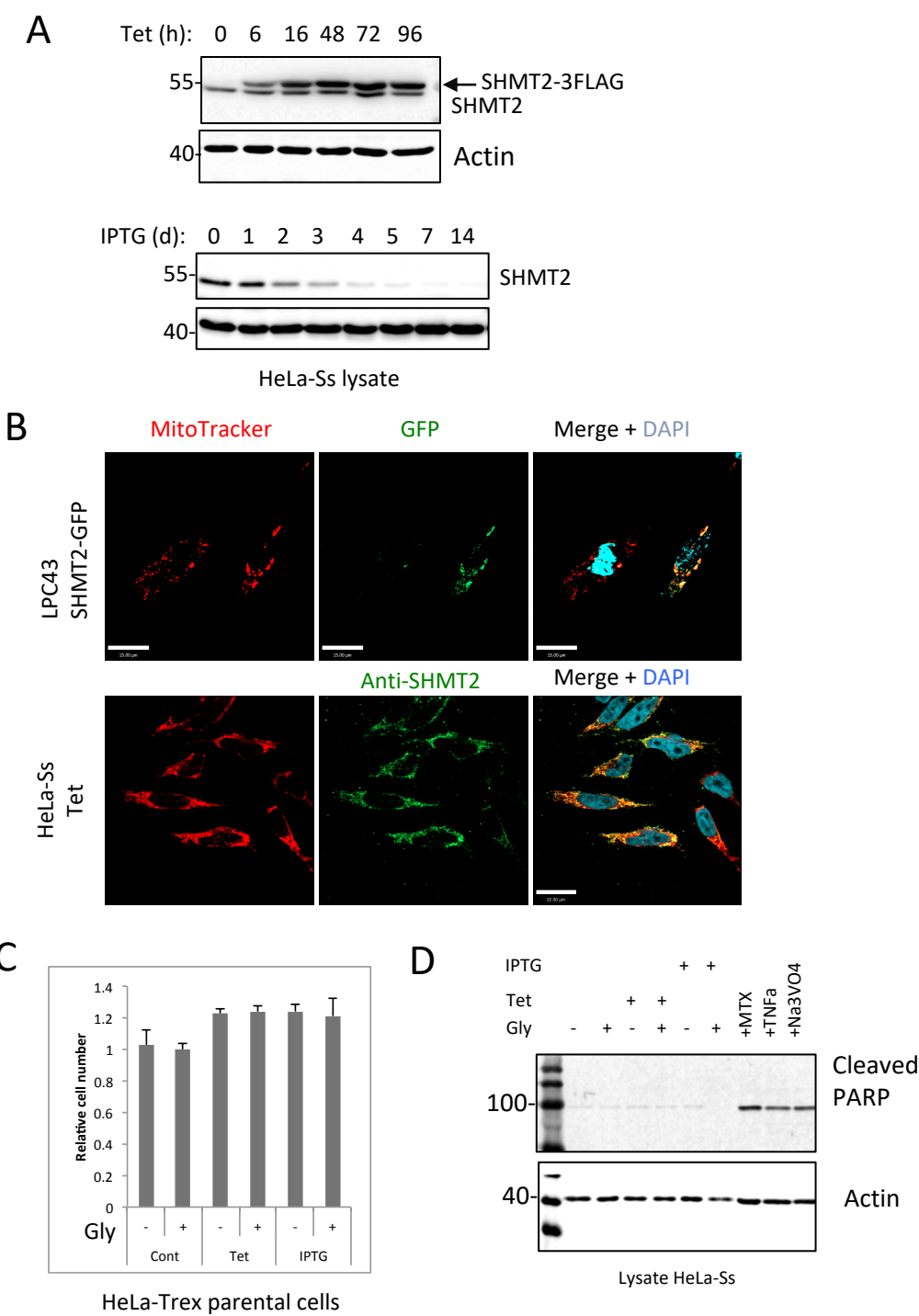

Supplement: S1 Fig — (A) WB analysis of whole cell lysates (WCL) from HeLa-Ss cells with Tet or IPTG treatments. HeLa-Ss cells were engineered for inducible ectopic expression of SHMT2-Flag (Up) and shRNA against SHMT2, which knocked down endogenous SHMT2 expression (Down). Results shown are representative of three independent experiments. (B) Immunofluorescence imaging of LPC43 cells transfected with SHMT2-GFP vector and HeLa-Ss cell with Tet treatment. After 48 h of SHMT2-GFP transfection, LPC43 cells were incubated with MitoTracker Red for 30 min in cell culture incubator and fixed. After 24 h Tet induction, HeLa-Ss cells were incubated with MitoTracker Red and fixed, which were then stained with anti-SHMT2 antibodies (green) and DAPI (blue for nuclear staining). Yellow color indicates co-localization. Scale bars are 15 μm. (C) Effect of Ted and IPTG on parent HeLa cell growth. HeLa cells were treated with Tet or IPTG for 3 d and cell number was measured by a crystal violet method and compared to cells grown in glycine supplemented medium. (D) Effect of SHMT2 expression and glycine depletion on apoptosis. Cleaved PARP is used as indicator of apoptosis and WB was used to analyze WCL from HeLa-Ss cells with indicated treatment. (PDF) [file pone.0237981.s003.pdf]

S2 Fig

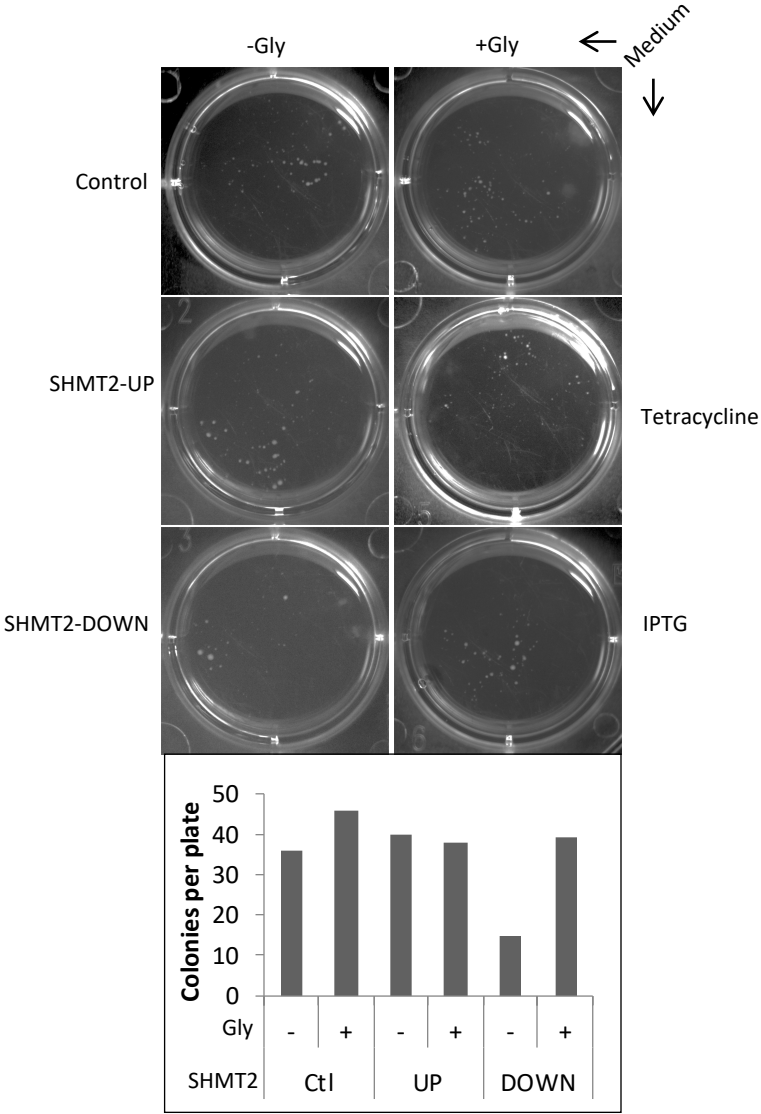

Supplement: S2 Fig — Soft agar assay was used to assess effect of target gene on anchorage independent growth. The conditions on cell culture were marked beside each well. Total numbers of colonies for a single representative experiment are shown in bar graphs below. (PDF) [file pone.0237981.s004.pdf]

A

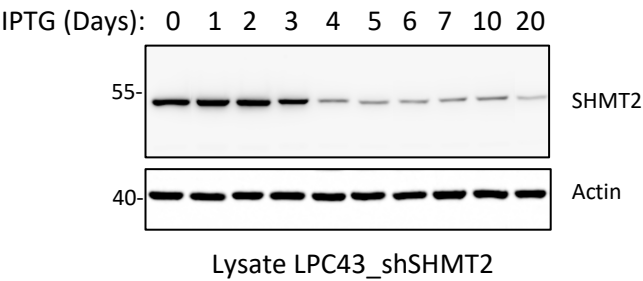

B

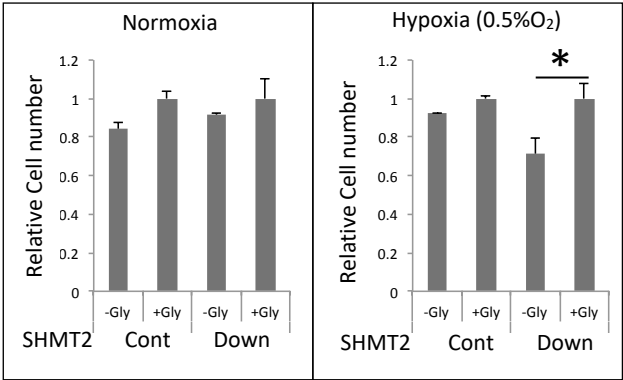

Supplement: S3 Fig — (A) Effect of IPTG inducible shRNA against SHMT2 in LPC43 cell. LPC43 is a human cell line derived from a NSCLC patient-derived xenograft (25). (B) LPC43 cell growth under normoxia and hypoxia conditions. LPC43 cells were treated with IPTG for 7 days and split on the same time as control cells, 3 d later the cell number was measured by a crystal violet method. Cells were tested in synthetic medium lacking glycine, and supplemented with dialyzed (glycine-free) serum (-Gly) or with addition of 100 mM glycine (+Gly). SHMT2 Down cells were treated with IPTG, therefore, had reduced SHMT2 expression. Control (Cont) cells were LPC43 cell without IPTG treatment. (PDF) [file pone.0237981.s005.pdf]

S4 Fig

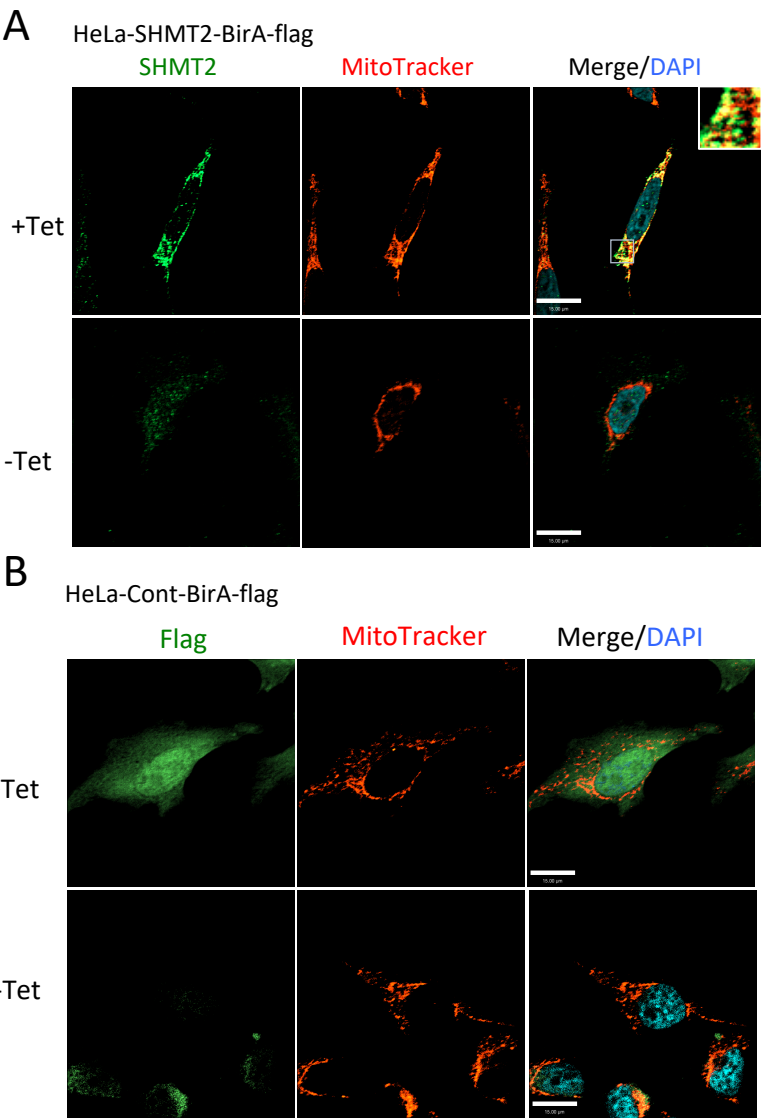

Supplement: S4 Fig — (A and B) Immunofluorescence imaging of SHMT2-BirA (A) and Cont-BirA (B) alone in HeLa cells. DNA encoding Flag-tagged BirA or SHMT2-BirA were stably incorporated into HeLa-Trex genomic DNA, and Ted treatment of cells induced their expression. After 24 h Tet induction, HeLa-SHMT2-BirA-Flag or HeLa-Cont-BirA-Flag cells were incubated with MitoTracker Red and fixed and then stained with anti-SHMT2 antibodies or anti-Flag antibodies (green). Yellow color indicates co-localization. Scale bars are 15 μm. (PDF) [file pone.0237981.s006.pdf]

A

HEK-293 + SHMT2-GFP

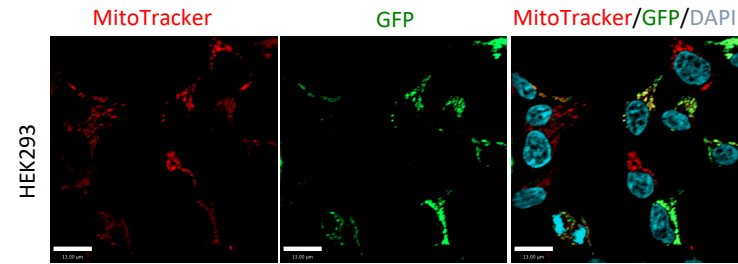

B

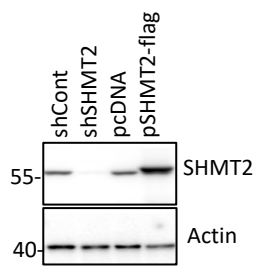

C

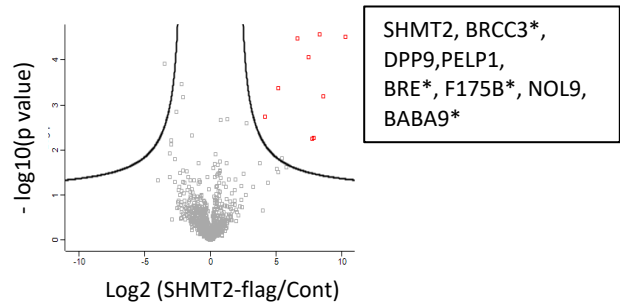

Supplement: S5 Fig — (A) SHMT2-GFP transient over-expression in HEK-293 cells. After 48 h of SHMT2-GFP transfection, HEK-293 cells were incubated with MitoTracker Red for 30 min in cell culture incubator and fixed. Yellow color indicates co-localization of mitochondrion maker (Red) and SHMT2 (Green). (B) Changes of SHMT2 expression in HEK-293 engineered cells. shRNA against SHMT2 was introduced into HEK-293 cell by lentivirus with puromycin selection; ectopic over-expression of Flag-tagged SHMT2 was introduced into HEK cells by pcDNA3 vector with G418 selection. WB analysis of WCL on stable cell lines was shown. (C) Volcano plot analysis of anti-Flag immunoprecipitation of SHMT2 over-expression cells from three biological repeats for each sample. Red dots indicate SHMT2 specifically associated proteins and * indicates proteins in BRISC complex. (PDF) [file pone.0237981.s007.pdf]

A  
Glycine

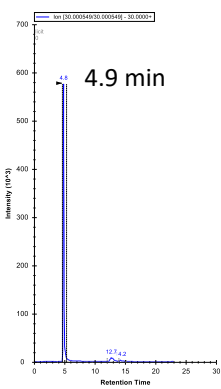

B

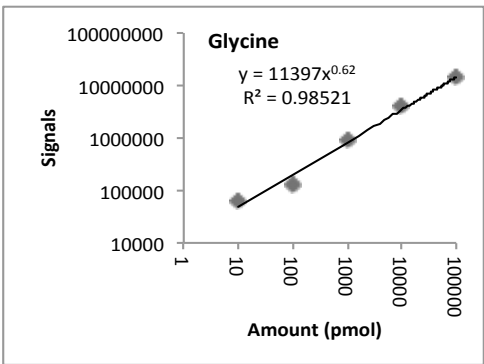

C

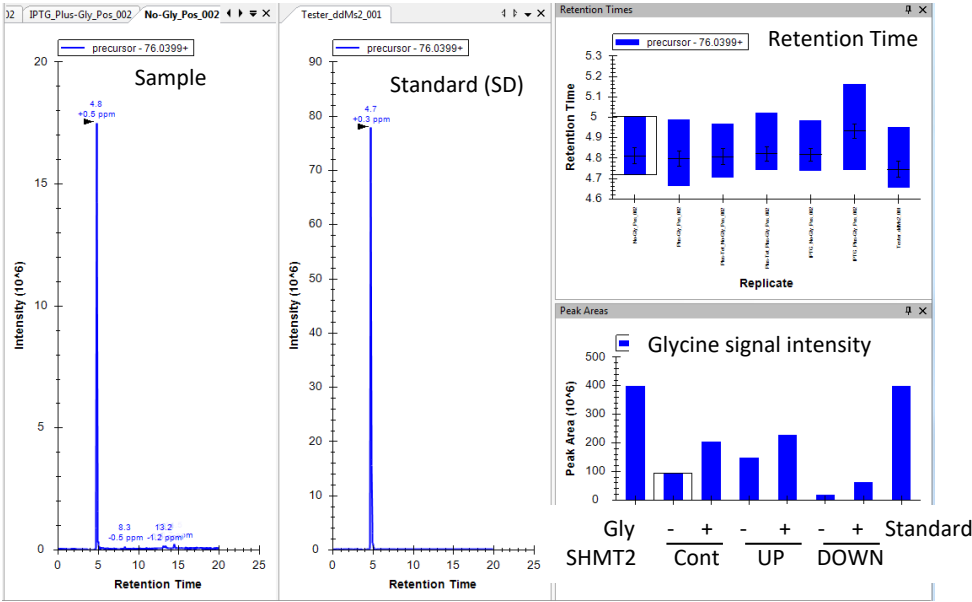

Supplement: S7 Fig — (A) A chromatograph of glycine standard measured by SRM and quantified by Skyline software. (B) Dose-response curve of a glycine standard measured with SRM. (C) Quantification of glycine in indicated samples. Left two panels are chromatographs of glycine from sample and standard. Right top panel is the alignment of retention time of samples and standards. Right bottom panel is the quantification result of indicated samples and standard. (PDF) [file pone.0237981.s009.pdf]

S8 Fig

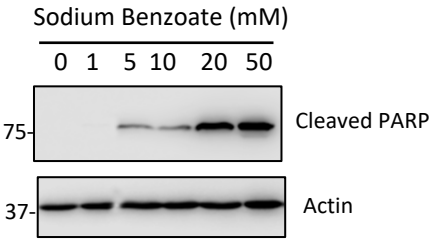

Supplement: S8 Fig — WB was carried out with indicated antibodies. Cleaved PARP is used as an indicator of cell apoptosis. (PDF) [file pone.0237981.s010.pdf]

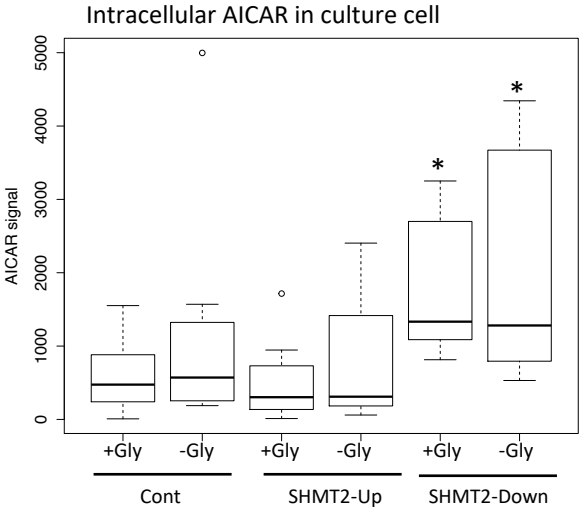

Supplement: S9 Fig — Targeted metabolite, AICAR, was extracted from HeLa-Ss cell in vitro and measured by SRM. Data were analyzed as described in Fig 5; * p < 0.05. (PDF) [file pone.0237981.s011.pdf]

S10 Fig

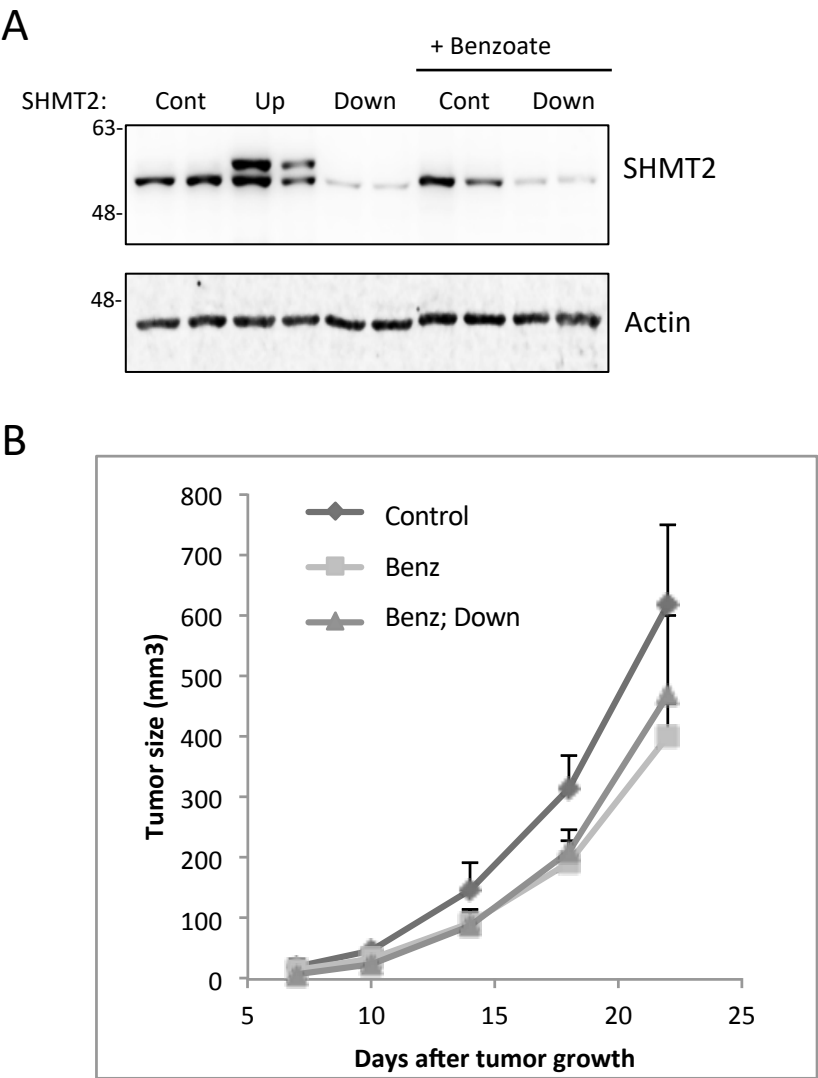

Supplement: S10 Fig — (A) SHMT2 expression in ten indicated tumor samples. Western blot was carried out with indicated antibody. (B) Effect of benzoate treatment on tumor growth in vivo. SCID mice bearing HeLa-Ss cell lines treated with Benz plus or minus shRNA-SHMT2 (Down), were monitored for tumor growth. The mean tumor volumes (± SD) for each group (n = 4 to 9) are shown. (PDF) [file pone.0237981.s012.pdf]

A

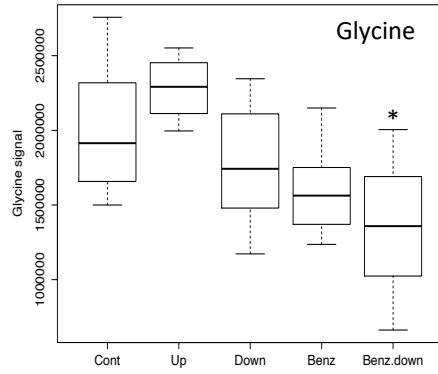

B

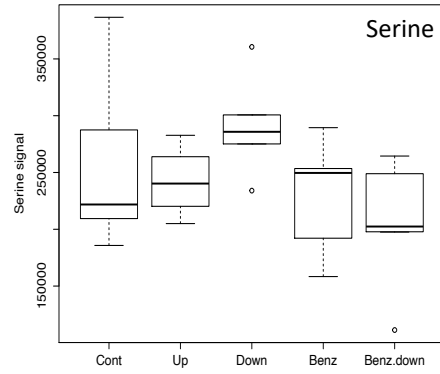

C

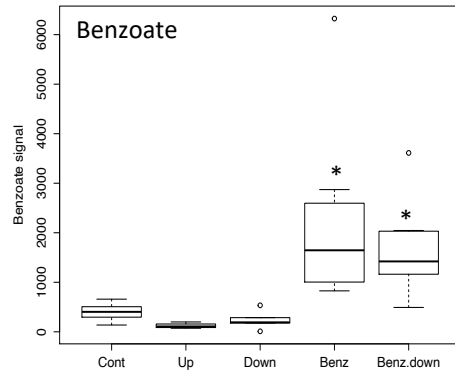

D

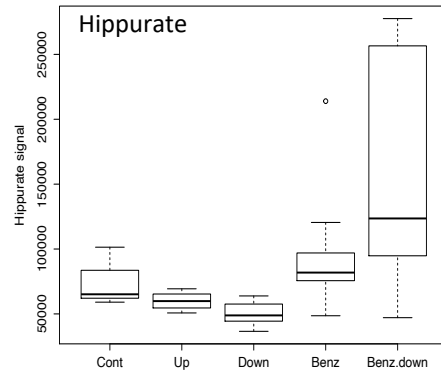

E

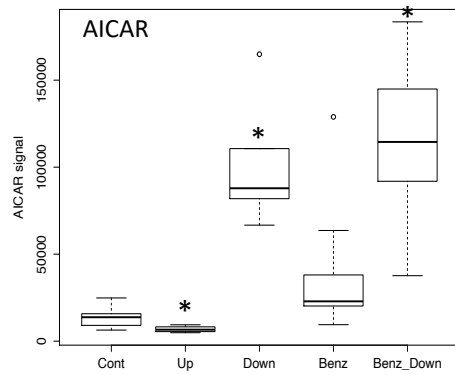

Supplement: S11 Fig — (A-E) Targeted metabolites were extracted from tumor and measured by SRM. Data were analyzed as described in Fig 5; * p < 0.05. (PDF) [file pone.0237981.s013.pdf]

S12 Fig

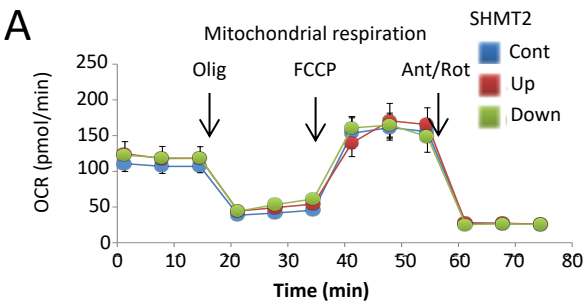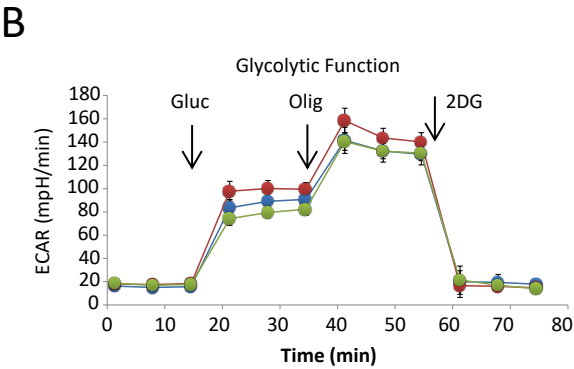

Supplement: S12 Fig — A Seahorse XFe96 Extracellular Flux analyzer was used to measure mitochondrial oxygen consumption rate (OCR) and extracellular acidification rate (ECAR). (A) Real time OCR of control (blue), SHMT2 over-expression (red) and knockdown (green) HeLa-Ss cells in mitochondrial stress tests. (B) Real time ECAR measurements of control, SHMT2 over-expression and knockdown HeLa-Ss cells in glycolysis stress tests. All data were presented as the mean ± standard deviation from 6–14 replicate wells per experiment. * p < 0.05 and ** p < 0.001 (Student’s t-test). (PDF) [file pone.0237981.s014.pdf]

S13 Fig

A

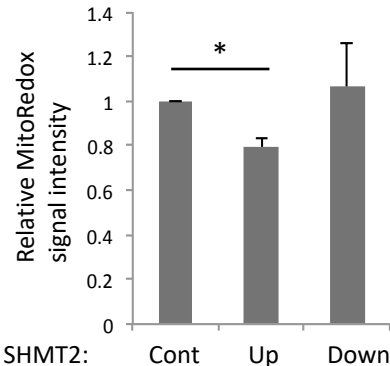

B

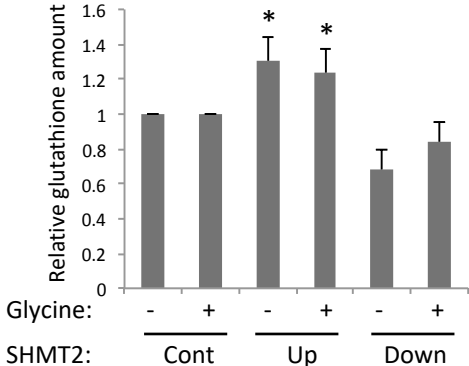

Supplement: S13 Fig — (A) Quantification of MitoTracker Red amount in indicated cells. HeLa-Ss cells were cultured in 96 cell plates, labeled with MitoTracker Red, and measured at 579/612 nm. (B) Relative amount of glutathione in living cells. Glutathione was measured with monochlorobimane (MCB) assay. * p<0.05 compared with control sample (Cont). (PDF) [file pone.0237981.s015.pdf]

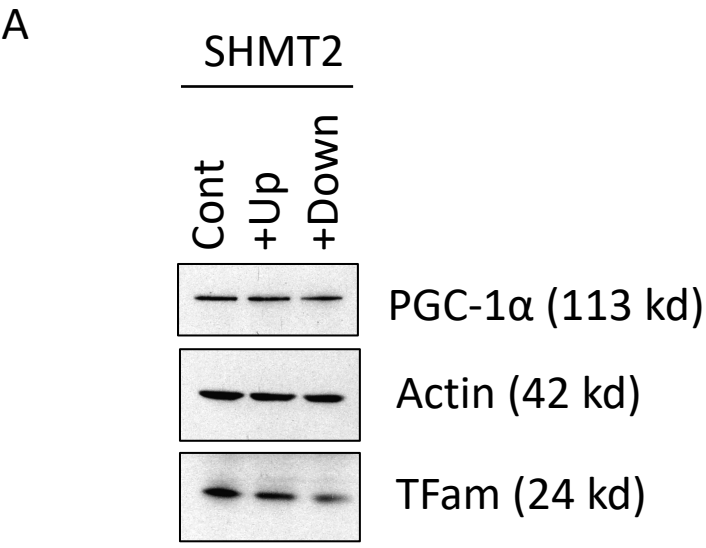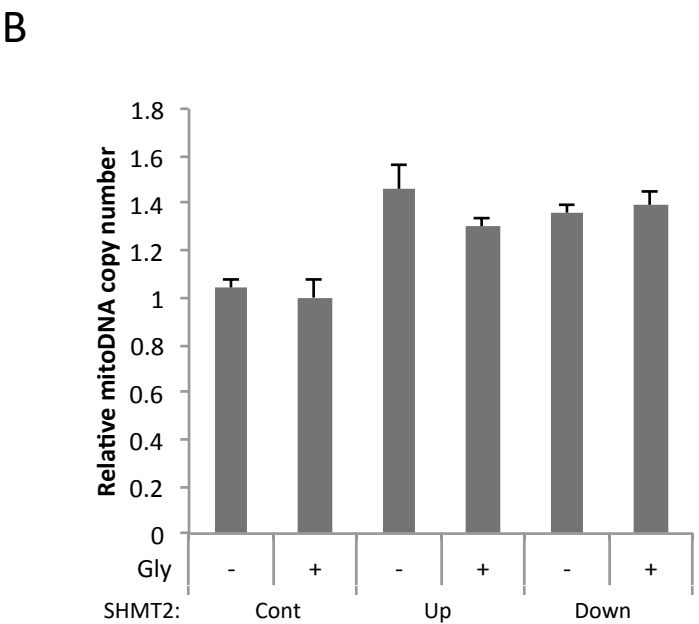

Supplement: S14 Fig — (A) WB analysis of two mitochondrial proteins (PGC-1α and TFam) with indicated antibodies. (B) Quantification of mitochondrion DNA copy number. Total DNA was extracted from indicated cells. Quantitative PCR with specific primers for entire D-loop of mtDNA DNA and β-actin were used to quantify DNA amounts as described previously [52]; relative amount are shown. (PDF) [file pone.0237981.s016.pdf]
