# Supplementary material for: Cancer proteome and metabolite changes linked to SHMT2
Source: PLoS One. 2020 Sep 9;15(9):e0237981. doi: 10.1371/journal.pone.0237981 (PMC7480864; doi:10.1371/journal.pone.0237981)
Supplement: S6 Fig — Functions of proteins are illustrated above the heatmap. Heatmap is the relative amount of proteins compared with control samples. Proteins with pink color (>1) indicates a relative abundance more than control; blue (<1) indicates less. (PDF) [file pone.0237981.s008.pdf]

S6 Fig

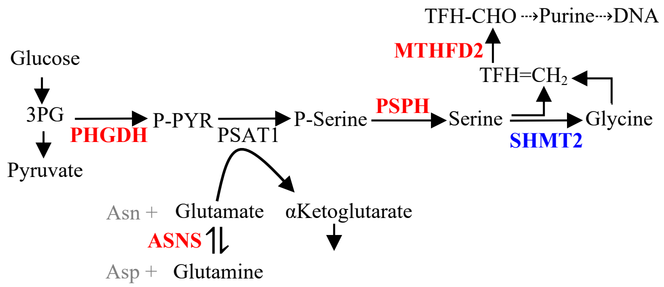

Protein expression level compared to control (Log2 ratio)

SHMT2:	in vitro Up	in vitro Down	in vivo Up	in vivo Down
SHMT2	15.27	0.65	2.59	0.38
PHGDH	1.09	1.06	1.09	0.95
GLS	1.03	1.04	1.05	0.97
ASNS	1.05	0.95	1.23	0.95
PSPH	1.05	0.97	1.15	0.89
CBS	1.06	0.92	1.18	0.95
PTDSS1	1.04	1.12	0.80	0.95
SHMT1	1.01	1.02	0.95	0.89
MTHFD1	1.04	1.06	1.07	1.12
MTHFD1L	1.02	0.93	1.05	1.14
MTHFD2	1.06	1.04	1.05	0.94
GCSH	0.54	0.60	0.61	0.71

Legend: pink = >1; blue = <1
